# Unraveling water monitoring association towards weather attributes for response proportions data: A unit-Lindley learning

**DOI:** 10.1371/journal.pone.0275841

**Published:** 2022-10-14

**Authors:** Paulo H. Ferreira, Anderson O. Fonseca, Diego C. Nascimento, Estefania Bonnail, Francisco Louzada

**Affiliations:** 1 Department of Statistics, Federal University of Bahia, Salvador, Bahia, Brazil; 2 Department of Mathematics, University of Atacama, Copiapó, Atacama, Chile; 3 Coastal Research Center, University of Atacama, Copiapó, Atacama, Chile; 4 Institute of Mathematical and Computer Sciences, University of São Paulo, São Carlos, São Paulo, Brazil; TDTU: Ton Duc Thang University, VIET NAM

## Abstract

Learning techniques involve unraveling regression structures, which aim to analyze in a probabilistic frame the associations across variables of interest. Thus, analyzing fraction and/or proportion data may not be adequate with standard regression procedures, since the linear regression models generally assume that the dependent (outcome) variable is normally distributed. In this manner, we propose a statistical model called unit-Lindley regression model, for the purpose of Statistical Process Control (SPC). As a result, a new control chart tool was proposed, which targets the water monitoring dynamic, as well as the monitoring of relative humidity, per minute, of Copiapó city, located in Atacama Desert (one of the driest non-polar places on Earth), north of Chile. Our results show that variables such as wind speed, 24-hour temperature variation, and solar radiation are useful to describe the amount of relative humidity in the air. Additionally, Information Visualization (InfoVis) tools help to understand the time seasonality of the water particle phenomenon of the region in near real-time analysis. The developed methodology also helps to label unusual events, such as *Camanchaca*, and other water monitoring-related events.

## 1 Introduction

Data acquisition related to natural resources is, day to day, more and more abundant, due to the miniaturization and reduction of data storage costs. Additionally, adopting the Internet of Things (IoT) technology helps to connect countless of decentralized devices and sustainability sources in benefits of analyzing to enhance planning, delivery, and efficiency of existing sources. These elements foment the decision-making performed on processing in near real-time, which remains a big challenge, given the need to configure systems to run every few minutes or hours, which causes them to process only the most recent features stored using robust data analysis tools.

Environmental Indicators (EI) play an important role in the sustainability in order to disseminate global environment statistics, based on the wide range of data sources, streamlining processes with IoT. Combined with Information Visualization (InfoVis), visual techniques provide support for specialists to visually summarize, explore, and reveal trends and patterns within data sets. Nonetheless, they are still in an early stage of development in many countries, and data are often sparse.

For instance, water source patterns may be seen as a combination of attributes (such as radiation, temperature, wind information, etc.) that impact directly this water proportion/rate. Moreover, in Atacama Desert, the water resources limitation demands extra need to unravel such dependent structure for water particles monitoring [1] and watershed estimation/forecasting.

In the case of rates and proportions processes, whereas the observed variable assumes values in the range (0, 1), there is a well-represented class of models, the unit distributions family, which deals with this type of sensor data, but are often univariate and not extended, in their inference, to some regression structures (see, e.g., [2–5]). Regression structures, in probabilistic modeling, can provide a flexible set of tools for examining such associations, while enabling, either, potentially confounding effects of other factors, or interaction effects for Statistical Process Control (SPC) tools [6].

This study considers a statistical model called unit-Lindley regression model [7], in which through the logit transformation, new variables can be incorporated into the parameter estimation, and the adequacy of some statistical association across those explanatory variables on the response variable can be verified. Furthermore, for the purpose of SPC, a new control chart is proposed targeting the water monitoring dynamic in Copiapó city, located in Atacama Desert (one of the driest non-polar places on Earth; see [8, 9]), north of Chile.

The upcoming contents of this paper are organized as follows. Section 2 describes the practical motivation. In Section 3, we present the unit-Lindley learning model and some of its basic properties (Subsection 3.1), as well as the novel control chart based on it (Subsection 3.2). Section 4 provides simulation studies designed to assess the performance of the proposed unit-Lindley regression control chart. Section 5 illustrates some findings towards the water particles monitoring in Atacama Desert through EI relationship. Finally, Section 6 concludes the paper with a few remarks and discussions on future studies.

## 2 Motivation

**Fig 1:**
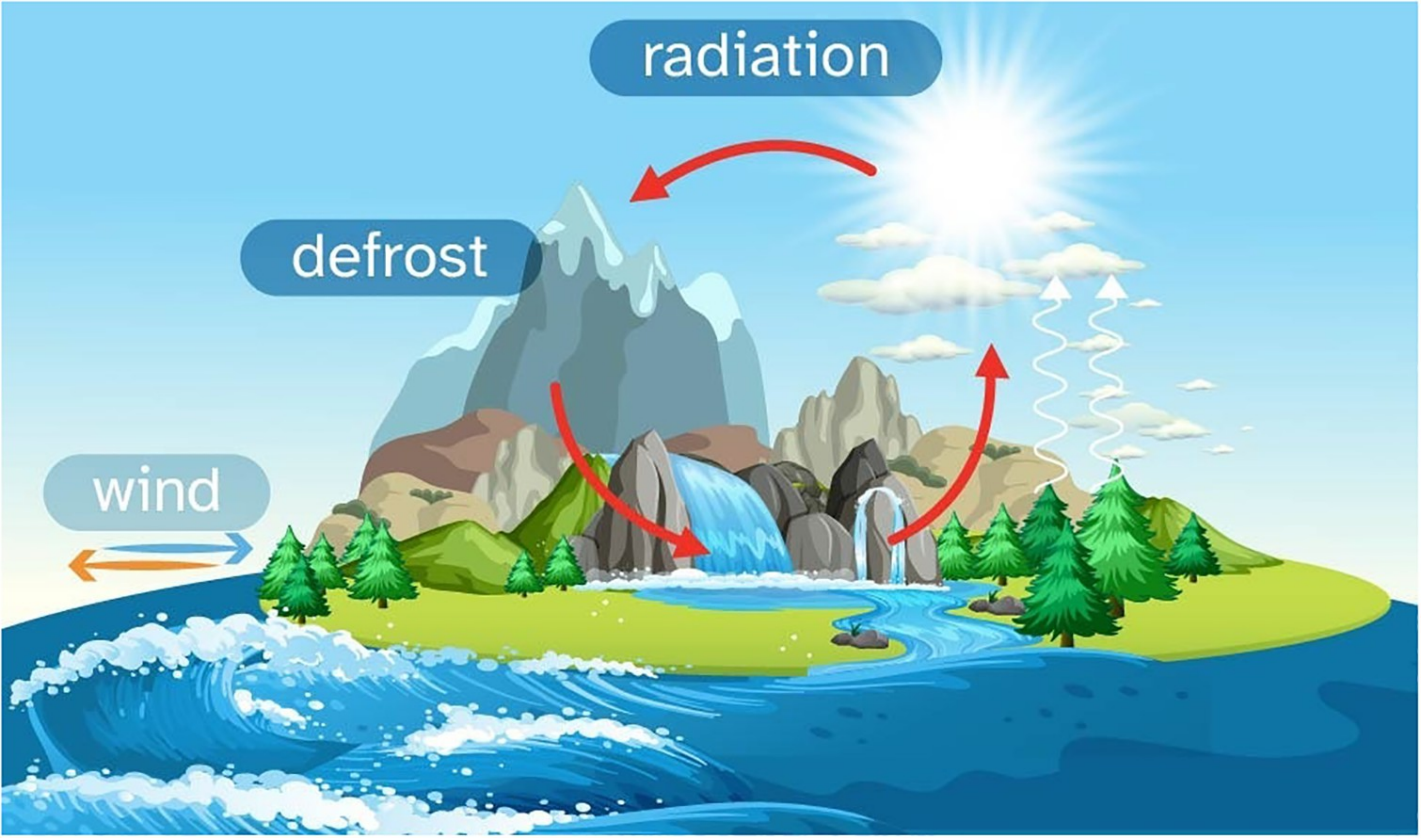
Water cycle sources (solid, liquid and vapor phases). Source: elaborated by the authors.

Water is the most precious resource that enables us to maintain the fauna and flora of a region, thus the existence of water resources is conditioned for its under-existence. An important water source from the Andean range is the cryosphere defrost, which generates water flows and underwater basins (Fig 1). Additionally, a phenomenon called *Camanchaca* occurs, whereas marine stratocumulus cloud banks that form in the Chilean coast blow as a passageway of “low clouds”, right after sunrise, in sequence for a couple of hours, creating a huge influence on the marks and infiltrating along the river valleys [10].

Relative humidity is the ratio of the partial pressure of water vapor in the air to reach an equilibrium in vapor pressure (of water), and there are three elements to be related with the water precipitation phenomenon: *temperature*, *wind movement*, and *solar radiation*. It is, therefore, the effective ratio of water content of the air in relation to the maximum water content that the air could contain (water in the form of vapor).

The maximum water content of the air depends directly on the temperature (the higher the temperature, the more water the air can contain, which is why when the temperature cools, the relative humidity increases, sometimes reaching saturation, which leads to the formation of fog or *Camanchaca*). Additionally, the wind moves the air masses and will, therefore, influence in their water content. The wind will also affect the evaporation of water, which will, again, modify the water content of the air masses (but directly linked to the moves of air masses). Finally, radiation affects the temperature and, therefore, the equilibrium in vapor pressure.

During precipitation, the falling water will partly evaporate, again modifying the water content of the air masses (but also decreasing the temperature since evaporation requires an energy input, drawn from the environment). The altitude from which precipitation is initiated is, therefore, important. The lower layers will gain water and lose temperature, but the cloud will lose water and warm up.

As for the *Camanchaca* phenomenon, fog often forms at sea level during the night, when the air temperature cools both because of the night and because of the contact with the cold waters of Humboldt Current [1]. The westerly winds, then, push these fog banks inland, preferably following the valleys, as the reliefs crossing implies in a loss of humidity by cooling (thus, a decrease in relative humidity, which potentially causes the fog to disappear).

The particular climatological conditions of the region often imply in a very low temperature inversion, normally present around 10,000 meters (m) over the Earth, but sometimes below 1,000 m in the Atacama region [11]. Due to the altitude, the temperature normally decreases till the tropopause (the boundary between the troposphere and the stratosphere), and then increases again in the stratosphere. In the Atacama region, the first temperature inversion around 1,000 m or less (and then another one, much more higher, that determines the tropopause), prevents the *Camanchaca* from going up in altitude (a temperature inversion is almost an insurmountable wall for air masses, with only the stratocumulus clouds having enough energy to cross this border).

## 3 Methodology

Human learning is majorly associated, directly and indirectly, with visual stimulation, whereas almost half of the neural tissue of the cognitive systems is related to pattern recognition through the vision [12]. The area of InfoVis aims to develop and apply visual representations towards the modeling and understanding of attribute values, relationships, and information extraction from data [13, 14]. InfoVis takes advantage of human cognition abilities to transform abstract data into visual information, since the effort to identify, interpret, and extract patterns is reduced when raw data is depicted in the form of graphical elements [15].

InfoVis techniques have been successfully applied to the analyses of different data sets (e.g., those related to business planning, social networks, climate, pollution, finances, criminal cases, etc.) [16–19] and in the identification of patterns and anomalies to be used as information to support decision-making [20], and can represent, both, unidimensional and multidimensional data (data sets with a large number of attributes related to each data record). InfoVis approach graphically represent information through computer systems, which show alternative data visions to describe their structures [21]. However, a large gap lies between the generation and storage of data, and the ability of analytical tools to process, organize and properly display the extracted information [22, 23].

**Fig 2:**
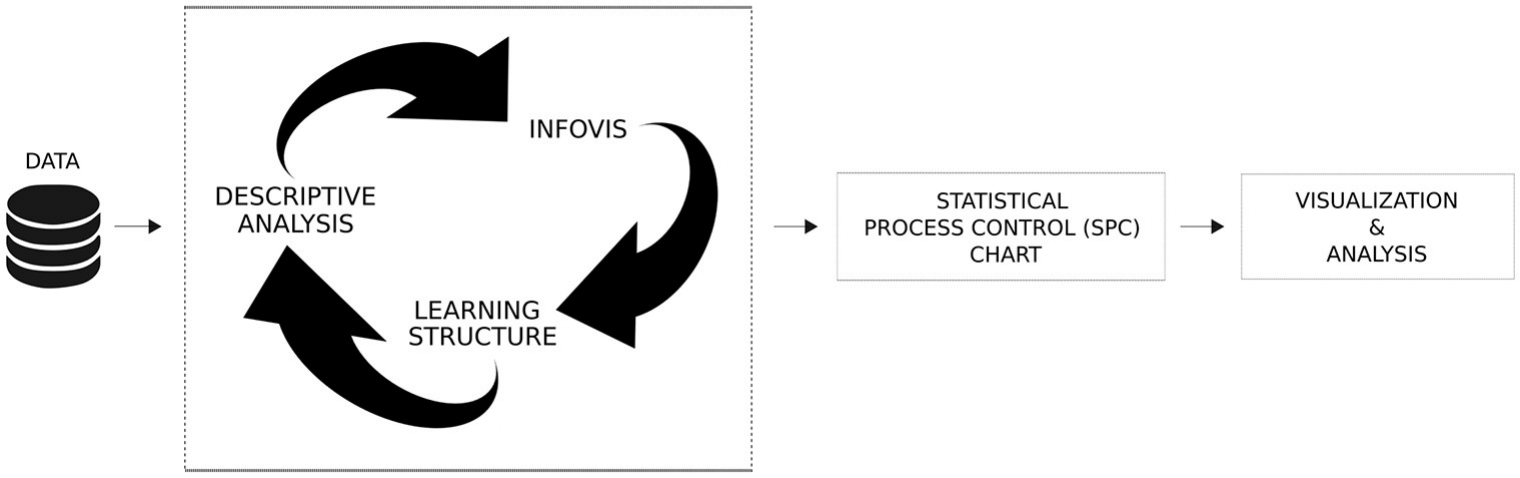
Visual representation of the adopted methodology.

In this manner, the following three-phase procedure is adopted towards the collected data: a Descriptive Analysis is combined with InfoVis, then an inference analysis is made through Learning Structure approach, with the unit-Lindley regression model (summarized in Fig 2).

In the following subsections, we will present, in details, the inferences related to the unit-Lindley distribution (Subsection 3.1), then its extension to SPC reasoning, as well as complementary regression (Subsection 3.2).

### 3.1 The unit-Lindley learning

Recently, [7] have introduced a one-parameter continuous probability distribution, defined by the interval (0, 1), called the unit-Lindley (UL) distribution. In this study, we shall consider a mean parameterized form of the UL distribution, also presented by the authors.

A random variable *Y* has a UL distribution with parameter 0 < *μ* < 1, denoted by *Y* ∼ UL(*μ*), if its cumulative distribution function is given by

$$\begin{array}{c} F\left(y|\mu \right)=1-[1-\frac{y(1-\mu )}{y-1}]\text{exp}\{-\frac{y(1-\mu )}{\mu (1-y)}\},\;\text{for}\;\;\;0<y<1. \end{array}$$

The corresponding probability density function (PDF) is

$$\begin{array}{c} f\left(y|\mu \right)=\frac{{\left(1-\mu \right)}^{2}}{\mu {\left(1-y\right)}^{3}}\text{exp}\{-\frac{y(1-\mu )}{\mu (1-y)}\},\;\text{for}\;\;\;0<y<1. \end{array}$$

If *Y* ∼ UL(*μ*), then the mean and variance of *Y* are given, respectively, by

$$\begin{array}{c} E\left[Y\right]=\mu \;\text{and}\;V\text{ar}\left[Y\right]=\mu [{\left(\frac{1}{\mu }-1\right)}^{2}\text{exp}\{\frac{1}{\mu }-1\}Ei(1,(\frac{1}{\mu }-1))-\frac{1}{\mu }+2]-\mu^{2}, \end{array}$$

in which $Ei\left(a,z\right)=\int_{1}^{\infty }y^{-a}e^{-yz}\;dy$ is the exponential integral function [24].

The quantile function of the UL(*μ*) distribution can be written as

$$\begin{array}{c} Q\left(p|\mu \right)=F^{-1}\left(p|\mu \right)=\frac{\frac{1}{\mu }+W_{-1}(\frac{\left(p-1\right)}{\mu }\text{exp}\{-\frac{1}{\mu }\})}{1+W_{-1}(\frac{\left(p-1\right)}{\mu }\text{exp}\{-\frac{1}{\mu }\})},\;\text{for}\;\;\;0<p<1, \end{array}$$ (1)

in which *W*_−1_ is the negative branch of the Lambert *W* function [25].

In a regression analysis, it is very common to model the mean of the response variable (the variable of interest) as a function of several other variables, also called explanatory variables or covariates. The UL distribution described above can be easily and promptly used in this context, as demonstrated by [7].

Let *Y*_1_, *Y*_2_, …, *Y*_*n*_ be *n* independent random variables, in which *Y*_*i*_ ∼ UL(*μ*_*i*_), for *i* = 1, 2, …, *n*. The so-called UL regression model is defined assuming that the mean of *Y*_*i*_ satisfies the following functional relation (linear predictor):

$$\begin{array}{c} g\left(\mu_{i}\right)=x_{i}^{⊤}\beta , \end{array}$$ (2)

in which $\beta ={\left(\beta_{1},\beta_{2},\ldots ,\beta_{k}\right)}^{⊤}\in R^{k}$ denotes a *k*-dimensional vector of regression coefficients (*k* < *n*), $x_{i}^{⊤}=\left(x_{i1},x_{i2},\ldots ,x_{ik}\right)$ represents the observations on *k* known covariates, and *g*(⋅) is a strictly monotonic and twice differentiable function that maps the interval (0, 1) into R (mean link function).

In this study, as in [7], we shall consider the logit link function, which ensures that the predicted mean stays within bounds (0, 1). Hence, the regression structure for *μ*_*i*_ is given by

$$\begin{array}{c} \text{logit}\left(\mu_{i}\right)=\text{log}(\frac{\mu_{i}}{1-\mu_{i}})=x_{i}^{⊤}\beta . \end{array}$$

Among other possible choices for the mean link function, we should mention the probit, cauchit, log-log and complementary log-log link functions (for these and other useful link functions, see [26]).

Under a classical inference approach, the unknown parameter vector ***β*** = (*β*_1_, *β*_2_, …, *β*_*k*_)^⊤^ can be estimated by maximizing the log-likelihood function:

$$\begin{array}{c} \ell \left(\beta \right)=\sum_{i=1}^{n}\ell \left(\mu_{i}\right),\; \end{array}$$

in which

$$\begin{array}{c} \ell \left(\mu_{i}\right)=2\text{log}\left(1-\mu_{i}\right)-\text{log}\left(\mu_{i}\right)-3\text{log}\left(1-y_{i}\right)-\frac{y_{i}\left(1-\mu_{i}\right)}{\mu_{i}\left(1-y_{i}\right)} \end{array}$$

and

$$\begin{array}{c} \mu_{i}={\text{logit}}^{-1}\left(x_{i}^{⊤}\beta \right)=\frac{\text{exp}\{x_{i}^{⊤}\beta \}}{1+\text{exp}\{x_{i}^{⊤}\beta \}}. \end{array}$$

Since the maximum likelihood (ML) estimator $\hat{\beta }$ of ***β*** cannot be expressed in closed form, we must resort to iterative methods, such as the Newton-Raphson and Broyden-Fletcher-Goldfarb-Shanno (BFGS) algorithms (for further details, see, e.g., [27]), to obtain the parameter estimates.

### 3.2 The unit-Lindley regression control chart

Let us start this subsection with a brief review about the UL control chart [1], which is useful to statistically monitor variables of rate or proportion type, that are independent and with no control variables being present. Subsequently, we will present the proposed UL regression control chart.

Suppose that a process (e.g., a manufacturing or business process) generates outputs according to a UL(*μ*) distribution, and the probability of false alarm (or equivalently, type I error) is given by *α*. Then, the lower control limit (LCL), centerline (CL) and upper control limit (UCL) of the UL control chart are defined as follows [1]:

$$\begin{array}{c} \text{LCL}=Q(\alpha /2|\mu ),\;\text{CL}=\mu ,\;\text{UCL}=Q(1-\alpha /2|\mu ), \end{array}$$

in which *Q*(.) is the quantile function presented in Eq (1).

It is worth mentioning that the use of the quantile function is justified by [28].

Nevertheless, the UL control chart does not consider situations in which the practitioner is required to impose a regression structure for the variable of interest. Our interest lies in cases in which the mean of the quality characteristic of interest (of rate or proportion type) is affected by control variables and can, then, be modeled as a function of them and unknown parameters.

Thus, considering the regression structure for *μ*_*i*_ defined in Eq (2) (e.g., using the logit link function), and a probability of false alarm equal to *α* (e.g., *α* = 0.0027, which corresponds to the standard three-sigma rule or Six Sigma program), we have that the non-constant (or observation-specific) LCL, CL and UCL of the proposed UL regression control chart are given by

$$\begin{array}{c} {\text{LCL}}_{i}=Q(\alpha /2|\mu_{i}),\;{\text{CL}}_{i}=\mu_{i},\;{\text{UCL}}_{i}=Q(1-\alpha /2|\mu_{i}), \end{array}$$

for *i* = 1, 2, …, *n*.

In practice, the ML estimator of *μ*_*i*_ is considered, with ${\hat{\mu }}_{i}=g^{-1}\left(x_{i}^{⊤}\hat{\beta }\right)$, in which $\hat{\beta }$ is the ML estimator of ***β***.

It is important to mention that there are two natural ways to visually represent and monitor this conditional structure: by adopting the *varying conditional mean* representation, or through its *residual* representation (that is, centralizing the process on zero by subtracting the expected value from the observed value).

## 4 Numerical evaluation

In this section, we conduct Monte Carlo (MC) simulations to assess and compare the performance of the proposed UL regression control chart with the existing beta regression control chart [28]. The performance comparisons are made in terms of the average run length (ARL), the median run length (MRL), and the standard deviation of the run length (SDRL). All statistical analyses were conducted using the R software version 3.6.3 [29].

The ARL is a popular measure used to assess the performance of control charts. The in-control and out-of-control ARLs are denoted as ARL_0_ and ARL_1_, respectively. The first one is defined as the average number of points plotted on the control chart until a signal occurs (that is, a single point falls beyond the control limits), assuming that the process is in control; whereas the second one represents the average number of observations that are taken before a mean shift is first detected when the process is out of control [30].

The MRL is the 50th percentage point of the run length (RL) distribution. In constrast to the ARL, the MRL is less affected by the skewness of the RL distribution [31]. The SDRL is a useful measure to estimate the dispersion of the RL distribution. Moreover, we will use the in-control (MRL_0_ and SDRL_0_) and out-of-control (MRL_1_ and SDRL_1_) versions of such metrics.

Let *Y* be the output of a process that follows a UL(*μ*) distribution, for instance. Also, let *μ*^(0)^ be the average of the process under control, and let *μ*^(1)^ be the average of the out-of-control process. Then, the ARL_0_, MRL_0_ and SDRL_0_ are defined as

$$\begin{array}{c} {\text{ARL}}_{0}=1/\alpha ,\;{\text{MRL}}_{0}=\text{log}\left(0.5\right)/\text{log}\left(1-\alpha \right),\;{\text{SDRL}}_{0}=\sqrt{\left(1-\alpha \right)/\alpha^{2}}, \end{array}$$

for $\alpha =P(Y\notin \left[\text{LCL},\text{UCL}\right]|\mu =\mu^{\left(0\right)})$. Whereas the ARL_1_, MRL_1_ and SDRL_1_ are given by

$$\begin{array}{c} {\text{ARL}}_{1}=1/\left(1-\gamma \right),\;{\text{MRL}}_{1}=\text{log}\left(0.5\right)/\text{log}\left(\gamma \right),\;{\text{SDRL}}_{1}=\sqrt{\gamma /{\left(1-\gamma \right)}^{2}}, \end{array}$$

for $\gamma =P(Y\in \left[\text{LCL},\text{UCL}\right]|\mu =\mu^{\left(1\right)})$.

In the usual Six Sigma program, *α* = 0.0027 and, therefore, ARL_0_ = 1/0.0027 ≈ 370, MRL_0_ = log(0.5)/log(1 − 0.0027) ≈ 256 and ${\text{SDRL}}_{0}=\sqrt{\left(1-0.0027\right)/0.0027^{2}}\approx 370$. This means, e.g. for the ARL_0_, that even though the process is in control, on average, a false alarm (incorrect out-of-control signal) will be generated at every 370 points [32]. On the other hand, low (i.e., close to one) values of ARL_1_ are desired, mainly for large-size shifts in the process mean.

### 4.1 General specifications

**Fig 3:**
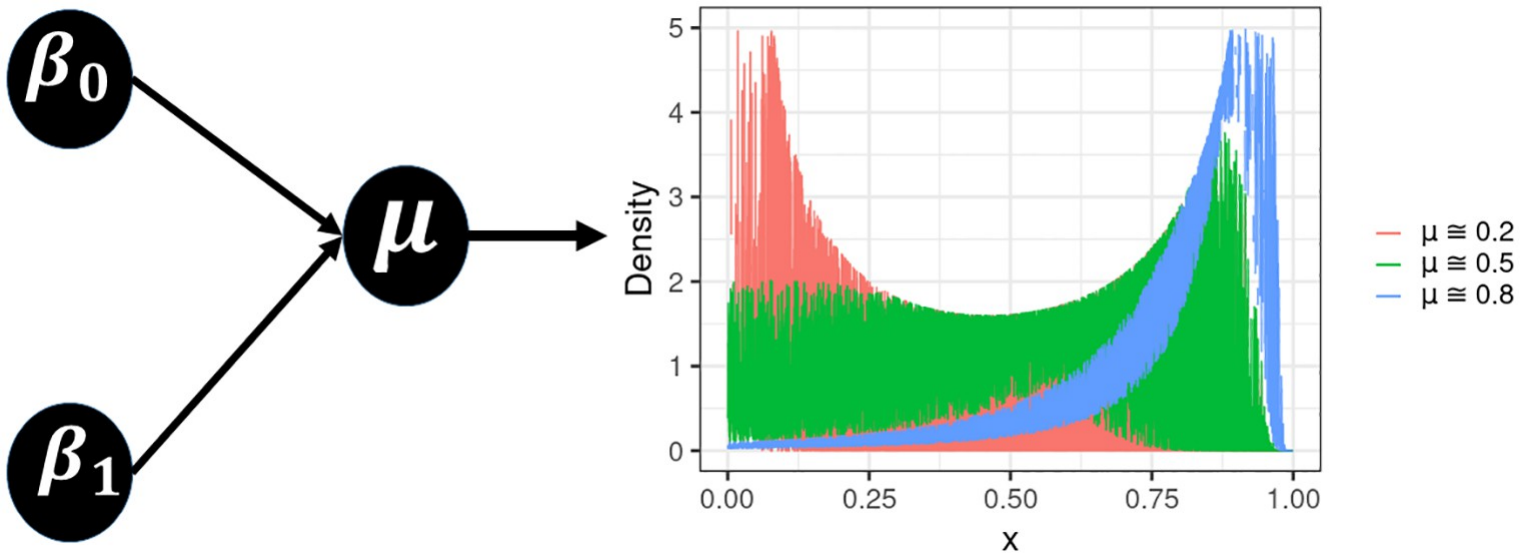
General description of the UL density function, followed by its illustration in different locations conditioned to the process mean parameter (*μ*).

**Fig 4:**
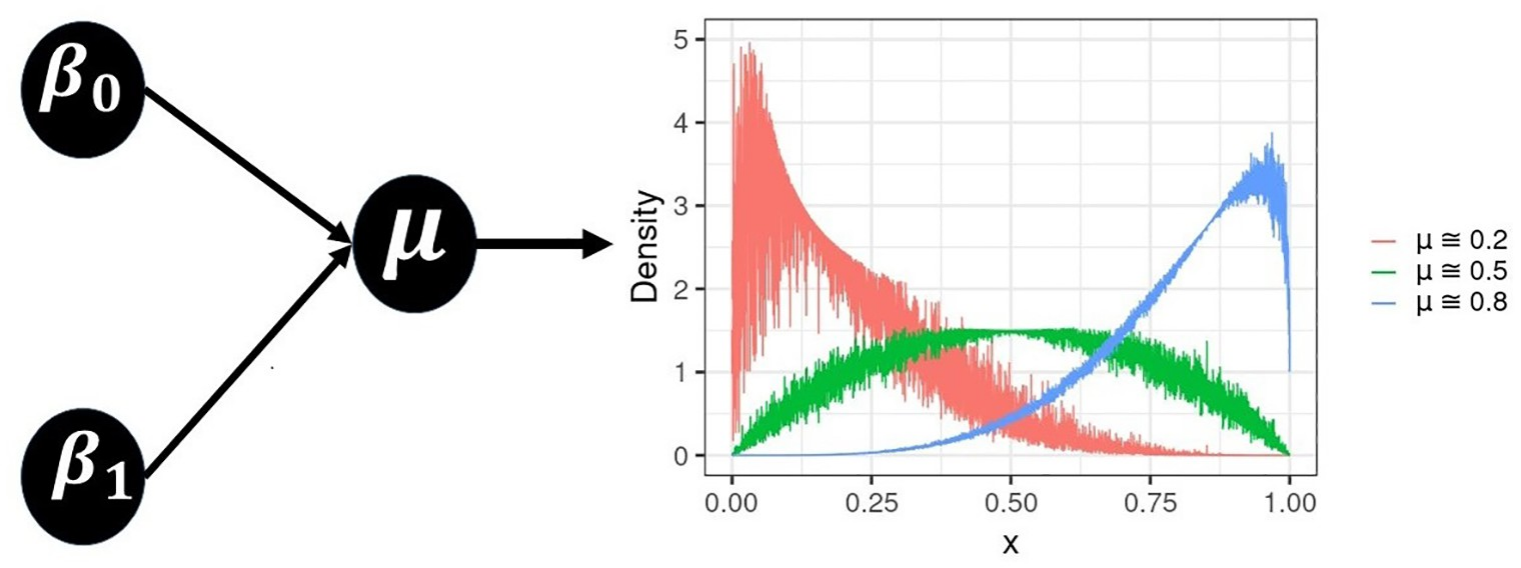
General description of the beta density function, followed by its illustration in different locations conditioned to the process mean parameter (*μ*).

Without loss of generality, we may consider UL and beta processes with (in-control) mean parameter: *μ*^(0)^ ≈ 0.2, 0.5 and 0.8, whose PDF plots are shown in Fig 3 (UL) and Fig 4 (beta), as well as two distinct values for the probability of false alarm: *α* = 0.1 (which corresponds to ARL_0_ = 10, MRL_0_ ≈ 6.579 and SDRL_0_ ≈ 9.487) and 0.01 (which corresponds to ARL_0_ = 100, MRL_0_ ≈ 68.968 and SDRL_0_ ≈ 99.499). We also use different sample sizes for each process: *n* = 100, 200, 500 and 1, 000. These *n* observations are devoted to Phase I or retrospective analysis (process parameter estimation → assessment of process stability → control limits establishment). Whereas n* = 5, 000 new observations are used to Phase II or prospective analysis (process monitoring → assessment of control chart performance). For the numerical evaluation, we consider 5,000 MC simulations (or replicates), which, according to [33], and also pointed out by other authors (e.g., [34]), is sufficient to obtain accurate results.

The generation of the data under control is based on the UL and beta regression models, with structure for the mean *μ*_*i*_ (around *μ*^(0)^) given by

$$\begin{array}{c} \text{log}(\frac{\mu_{i}}{1-\mu_{i}})=\beta_{0}+\beta_{1}x_{i}, \end{array}$$

in which the values of the single covariate are drawn from the Uniform(0, 1) and Normal(1, 0.01) distributions, for the UL and beta processes, respectively, that is, $X_{i}\overset{\text{iid}}{∼}\text{Uniform}\left(0,1\right)$, for *i* = 1, 2, …, *n*, *n* + 1, …, *n* + n*, when the true data-generating process is UL distributed, and $X_{i}\overset{\text{iid}}{∼}\text{Normal}\left(1,0.01\right)$, for *i* = 1, 2, …, *n*, *n* + 1, …, *n* + n*, when the true data-generating process is beta distributed. Here, the abbreviation *iid* stands for “independent and identically distributed”. The parameter values for the mean structure of each regression model are presented in Table 1 (UL) and Table 2 (beta).

**Table 1:** Parameter values for scenarios considered in the simulation, when the true data-generating process is UL distributed (in-control condition).

Scenario	*β*_0_	*β*_1_	Characteristic
1	1.00	-6.16	*μ* ≈ 0.2
2	1.00	-2.00	*μ* ≈ 0.5
3	1.00	0.77	*μ* ≈ 0.8

**Table 2:** Parameter values for scenarios considered in the simulation, when the true data-generating process is beta distributed (in-control condition).

Scenario	*β*_0_	*β*_1_	Characteristic
1	1.00	-2.39	*μ* ≈ 0.2 and *σ* ≈ 0.37
2	1.00	-1.00	*μ* ≈ 0.5 and *σ* ≈ 0.45
3	1.00	0.40	*μ* ≈ 0.8 and *σ* ≈ 0.38

For the estimation of beta regression model parameters, the R package gamlss [35] is used.

Finally, to compute the ARL_1_, MRL_1_ and SDRL_1_, we consider shifts at different levels, representing percentage decreases and increases *p* in the process mean. The assumed levels are: *p* = 1% (down-shifted mean: *μ*^(1)^ ≈ 0.198, 0.495 and 0.792; up-shifted mean: *μ*^(1)^ ≈ 0.202, 0.505 and 0.808) to 10% (down-shifted mean: *μ*^(1)^ ≈ 0.18, 0.45 and 0.72; up-shifted mean: *μ*^(1)^ ≈ 0.22, 0.55 and 0.88) and 20% (down-shifted mean: *μ*^(1)^ ≈ 0.16, 0.4 and 0.64; up-shifted mean: *μ*^(1)^ ≈ 0.24, 0.6 and 0.96). The parameter values for the mean structure of each regression model are shown in Table 3.

**Table 3:** Parameter values for scenarios considered in the simulation (out-of-control condition).

UL		Beta		Characteristic
*β*_0_	*β*_1_	*β*_0_	*β*_1_	
0.48	-5.77	0.58	-2.26	*μ* ≈ 0.160
0.70	-5.89	0.91	-2.41	*μ* ≈ 0.180
0.91	-6.08	0.97	-2.37	*μ* ≈ 0.198
0.96	-6.11	1.02	-2.37	*μ* ≈ 0.202
1.18	-6.30	1.06	-2.35	*μ* ≈ 0.220
1.46	-6.57	1.35	-2.50	*μ* ≈ 0.240
0.47	-1.76	0.46	-0.87	*μ* ≈ 0.400
0.76	-1.91	0.67	-0.87	*μ* ≈ 0.450
1.05	-2.11	1.00	-1.02	*μ* ≈ 0.495
1.12	-2.16	1.07	-1.05	*μ* ≈ 0.505
1.48	-2.43	1.33	-1.14	*μ* ≈ 0.550
1.95	-2.88	1.69	-1.28	*μ* ≈ 0.600
0.37	0.40	0.37	0.22	*μ* ≈ 0.640
0.68	0.50	0.70	0.25	*μ* ≈ 0.720
0.99	0.67	1.02	0.32	*μ* ≈ 0.792
1.05	0.76	0.98	0.47	*μ* ≈ 0.808
1.39	1.22	1.44	0.57	*μ* ≈ 0.880
1.27	5.35	1.13	2.12	*μ* ≈ 0.960

### 4.2 Simulation results

The results obtained with MC simulations, performed for each situation studied, are available in S1 Appendix. In short, such results seem to indicate (despite some slight to moderate discrepancies between the theoretical/target values of the performance measures, and the values calculated through simulations in some cases, which are, in fact, expected due to the effect of parameter estimation on control chart properties; see, for instance [36–38]) a good performance of the proposed regression control chart, mainly when the true data-generating process is UL distributed, and with increases in the process mean (this latter finding is in agreement with the results of [1]). In the cases in which data are generated from the beta distribution, the UL regression control chart tends to show slightly higher false alarm rates, whereas performing reasonably well when changes (increases and decreases) in the process mean occur. Once again, it is worth pointing out that the proposed regression control chart has its basis on a distribution with a single parameter (and, thus, more straightforward than the two-parameter beta distribution).

## 5 Application

The relative humidity of the air in the city of Copiapó, Chile, is particularly interesting to be monitored given that it is located in the heart of Atacama Desert, and also because it is an important northern Chilean city. The main economic source is related to the extraction of minerals and agriculture, both demanding great volume of water sources. Nevertheless, a natural water particle flux happens periodically given the geolocation of this city (placed in a valley, and about 60 kilometers open-field from the Chilean coast).

As a consequence, water events monitoring is needed, in relation to other natural phenomena, thus creating an opportunity to adopt the UL regression control chart for real data source.

The data set adopted in this study was acquired from the *Dirección General De Aeronáutica Civil, Dirección Meteorológica de Chile—Servicios Climáticos*, which provides several sets related to natural sources in Chile. Additionally, the water relative humidity-related *Wind*, *Temperature*, *Solar Radiacion*, and *Humidity Pressure* sets of the studied city. All records were taken from January 1st, 2019 to June 30th, 2021 (per minute), period in which some missing data are noticeable, resulting in a total of 1,237,596 data points. After data wrangling, we obtained a unified set containing 12 covariates: six related to the Wind dimension (*ddInst*, *ffInst*, *dd02Minutos*, *ff02Minutos*, *dd01MinutosMax*, *ff01MinutosMax*), two related to Temperature (*ts*, *td*), one related to Radiation (*radiacionGlobalInst*), and three related to Humidity (*hr*, *p0*, *qff*). The adopted data set is available at: https://doi.org/10.34740/KAGGLE/DSV/4051087, while the developed R script is available at: https://github.com/ProfNascimento/ULreg.

**Fig 5:**
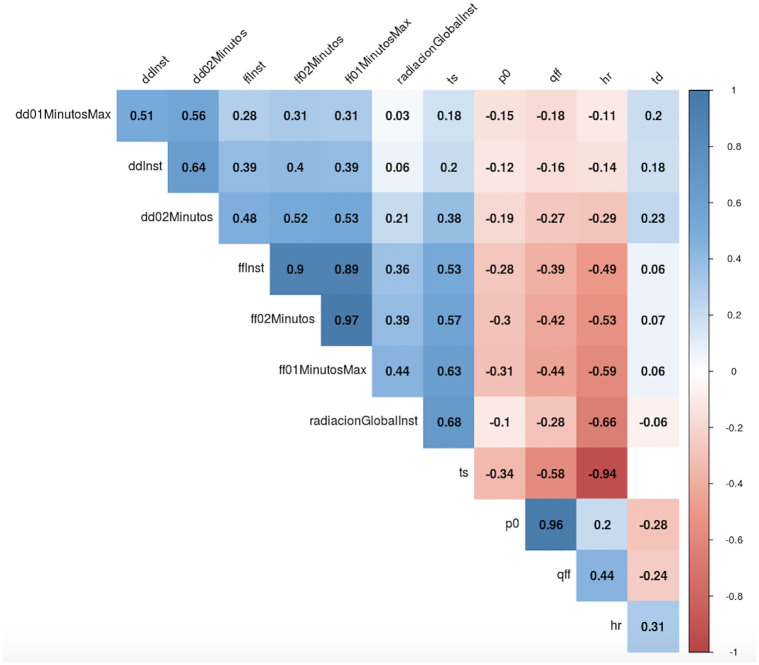
Correlation plot across weather variables.

Since all variables are continuous, as a first relation metric, we used the Spearman’s rank correlation coefficient. Fig 5 shows that, considering the relativity humidity (*hr*) variable, in module, the most associated covariates considering the same time period per dimension were: current temperature (*ts*), wind speed (*ffInst*), and solar radiation (*radiacionGlobalInst*). Moreover, in the Wind dimension, in module, the greatest correlated variable was *ff01MinutosMax*, thus highly positive related to the instantaneous speed (*ffInst*).

The first step before starting to seek some association across these explanatory variables (*X*’s), was to perform a multicollinearity checking. After adjusting an Ordinary Least Squares (OLS) model, three variables (*p0*, *qff*, *ts*) presented high Variance Inflation Factor (VIF), greater than 500 units in module, and were then excluded. Additionally, a presence of three clusters was noticed related to the response of the Wind, Temperature, and Radiation. Based on the expert knowledge, the selected variables to represent those clusters, and might be related to the relative humidity (theoretically), were the instantaneous wind speed (*ffInst*), instantaneous solar radiation (*radiacionGlobalInst*), and 24-hour temperature variation (*td*).

**Fig 6:**
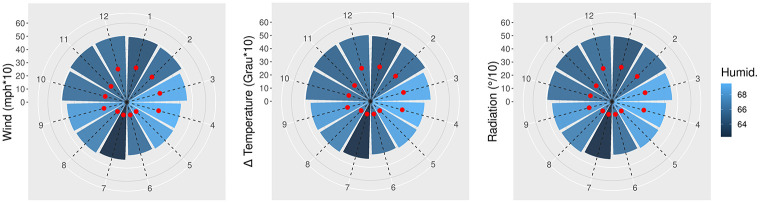
Radar plots: monthly wind variation (left-hand panel), 24-hour temperature variation (middle panel), and monthly solar radiation (right-hand panel). The red dots represent the minimum relative record for each month.

Looking closer into the instantaneous wind speed variable across time, Fig 6 depicts the monthly dynamic of this variable (left-hand panel), as well as the dynamic related to the instantaneous temperature (middle panel), and the solar radiation (right-hand panel). The lowest average records were from May to August, and the highest from December to February for all of these variables.

**Fig 7:**
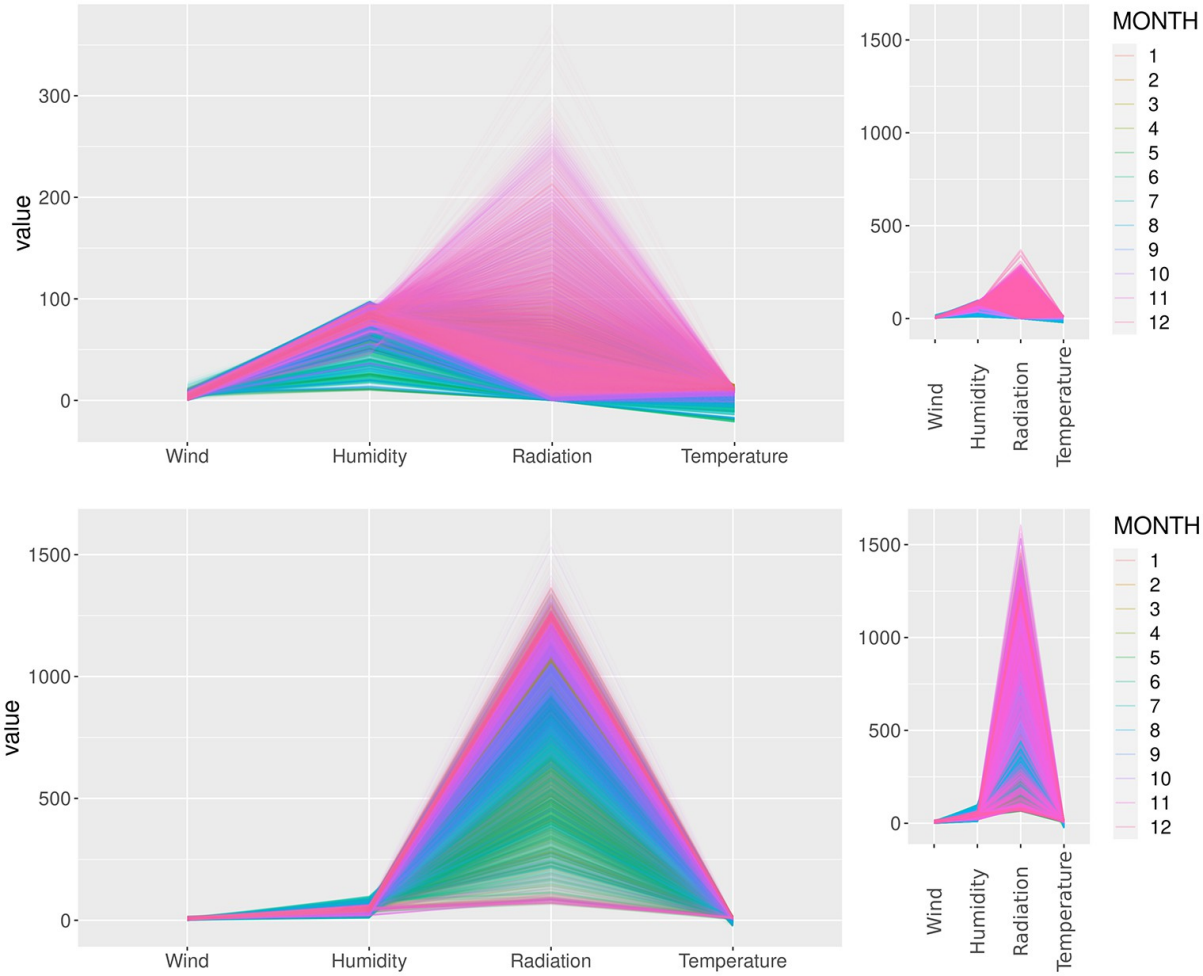
Parallel plots describing the records variation throughout time and across variables. The top graphics are related with Phase I, at 10 a.m., and, the bottom graphics, at 4 p.m. The summer period months (mostly pink tones) present great range of records across weather variables (wind, 24-hour temperature variation, solar radiation and relative humidity).

Fig 7 displays the variables’ multidimensional relation, through the parallel plot, in which each line is a day-related record. Specifically, the top graphics are related to 10:00–10:01 a.m. (first minute), and the bottom graphics to 4:00–4:01 p.m. (first minute). Moreover, the bright colors are related to November until March (seasons in which the days receive sunlight earlier). For instance, higher values are noticeable in the summer time, during the morning (given the sunlight incidence), and vary more throughout the year in the afternoon.

The theoretical model adopted for this problematic was:

$$\begin{array}{cc} & \text{Relative}\;{\text{Humidity}}_{i}∼\text{UL}\left(\mu_{i}\right),\;\text{in}\;\text{which}\\ \text{logit}(\frac{\mu_{i}}{1-\mu_{i}})= & \;\beta_{0}+\beta_{1}\;\text{Wind}\;{\text{Speed}}_{i}+\beta_{2}\;\Delta {\text{Temperature}}_{i}+\beta_{3}\;\text{Solar}\;{\text{Radiation}}_{i}, \end{array}$$

for *i* = 1, 2, …, 1, 207, 079 (Phase I).

Table 4 shows the estimates related to the three EI, which play an important role in the predictability, and association, of relative humidity. Since the link function related to the UL regression model is the logit function, further interpretation of *β* parameters requires to be transformed before. That is, for a unit increase in wind speed, on average, a change of exp{−0.0695*X}/(1 + exp{−0.0695*X}) → *X* = 1 ≈ 0.483 in the mean of the relative humidity, or the odds rate is to be expected. It is important to mention that a fraction contribution can also be adopted (based on proportion), since the response variable is a unit.

**Table 4:** UL regression model adjusted for relative humidity data.

	Estimate	Std. error	*t* stat	*p*-value
Intercept	0.7103	0.0026	301.5	<0.0001
Wind Speed	-0.0695	0.0003	-259.7	<0.0001
ΔTemperature	0.0585	0.0002	266.7	<0.0001
Solar Radiation	-0.0013	<0.0001	-665.1	<0.0001

**Fig 8:**
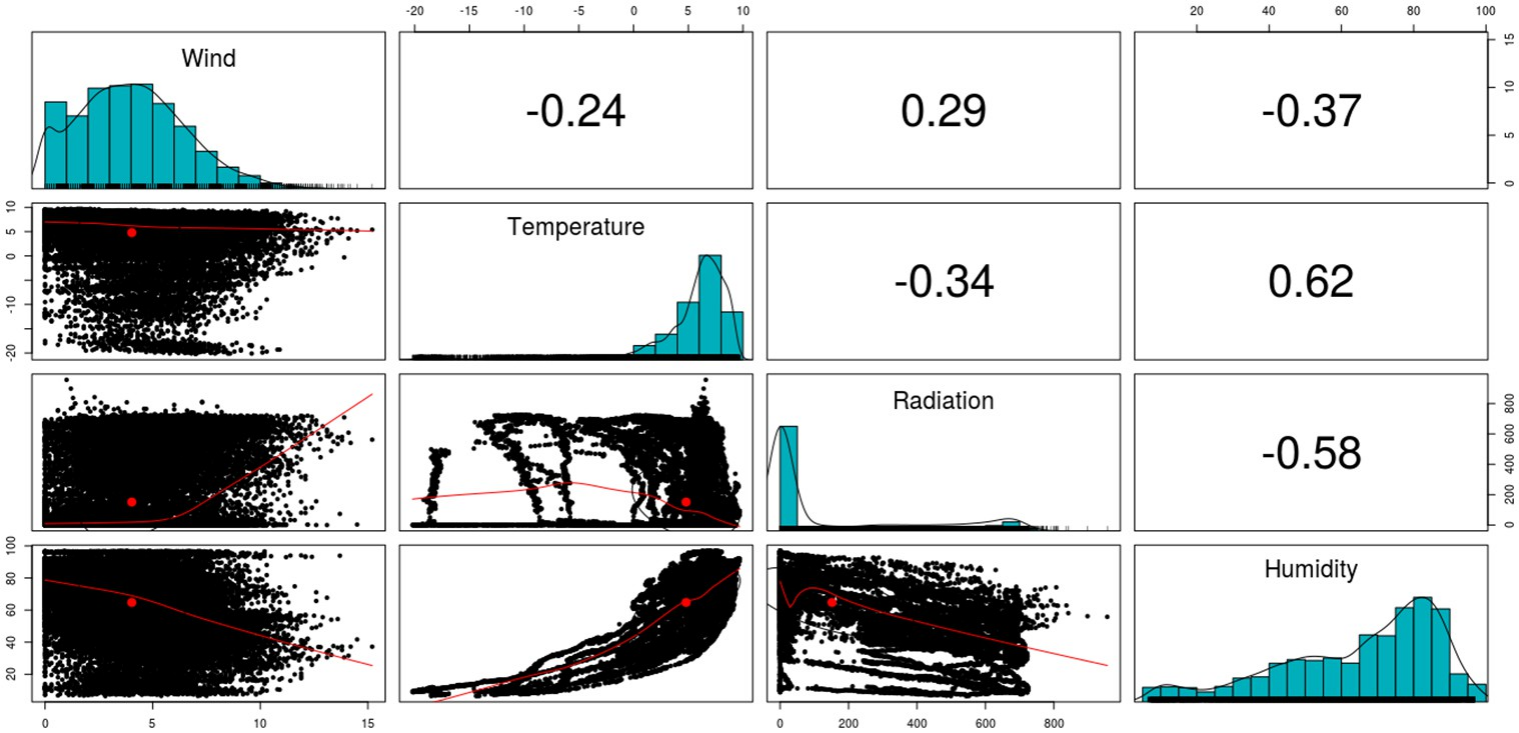
Pairwise representations adopting the Phase II observations, and their Spearman correlations.

Thus, through the use of Phase II observation points, that is, the last 23 days, it is also possible to confirm the similarity across the association obtained from the UL regression results from Phase I. Fig 8 shows the pair relation across the studied variables, from which negative Spearman’s rank correlation coefficients were obtained across Relative Humidity and Solar Radiation, and Relative Humidity and Wind Speed (similar to the estimated *β* coefficients from the UL regression model).

The historical data were captured per minute, and the relative humidity, for Phase II, was observed during June 9th, 7:21 p.m. and July 1st, midnight. The total number of observation points was of 30,517, and the adoption of the UL regression model enables to estimate the process mean varying in time. Table 5 summarizes the expected air humidity, per minute, and its variation in Copiapó city. Considering a Six Sigma policy supervision, on average per minute, the relative humidity fluctuation is centered in 62.1%, with range between 18.2% and 77.9%. The maximum that could be observed was up to 94.1% of humidity (thus, some influx of water particles from the ocean happens, even though the analyzed city is placed at 350 m from the sea level and no elevation is placed between the coast and this city).

**Table 5:** Relative humidity’s summary statistics obtained from the UL regression analysis, given time-varying SPC, for its expected mean and boundaries.

	LCL	CL (*μ*)	UCL
Minimum	0.014	**0.182**	0.436
1st Quartile	0.115	0.545	0.833
Median	0.223	0.669	0.898
Mean	0.207	**0.621**	0.863
3rd Quartile	0.289	0.718	0.918
Maximum	0.399	**0.779**	0.941

**Fig 9:**
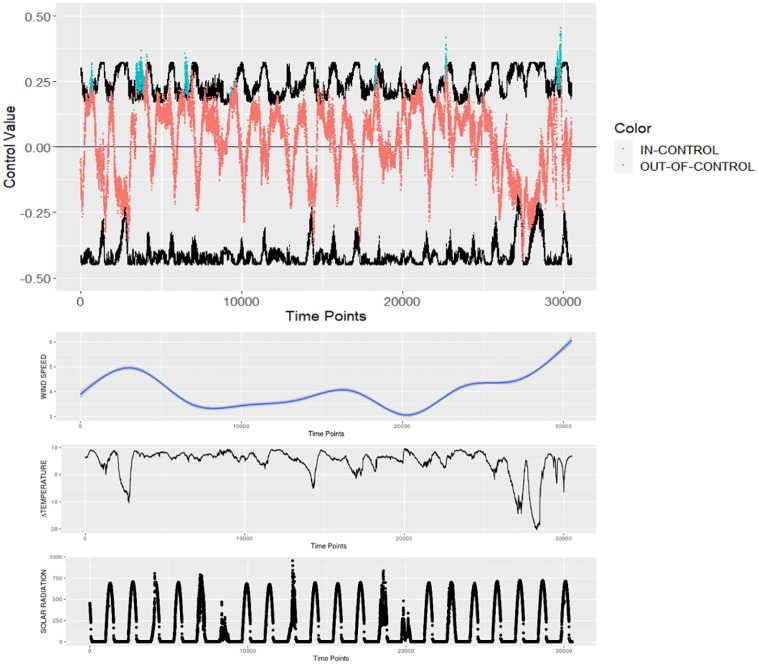
UL regression control chart (Phase II) adopting the residual representation (top panel), and the covariates’ dynamic plots (lower panels).

Moreover, the obtained SPC boundaries help to detect some massive water flux events, like *Camanchaca*, revealed by their discrepancy. Fig 9 shows the control chart in which the minutes when this suddenly discrepant water-related event started (top graphic, in blue colors), and the covariates’ response during this event (lower graphics).

We also compared the performance of the proposed approach with the one presented in [28]. That is, we also built control charts based on the beta regression model with constant and varying dispersion parameter (hereafter, beta 1 and beta 2 models, respectively). Note that the beta 1 model was already considered in Section 4. First, a performance comparison of the three models (UL, beta 1 and beta 2), in terms of Akaike Information Criterion (AIC) [39], Bayesian or Schwarz Information Criterion (BIC) [40], and Root Mean Squared Error (RMSE), is presented in Table 6. Observe that these models are practically equivalent in terms of RMSE, although considering the AIC and BIC criteria, the beta 2 model performs the best (i.e., it provides the best fit). The complete estimation results for the beta models, as well as the corresponding control charts, are shown in S2 Appendix.

**Table 6:** Performance comparison of the selected regression models using AIC, BIC and RMSE (Phase I data).

	AIC	BIC	RMSE
UL	-1,296,760	-1,296,656	0.129
Beta 1	-1,727,663	-1,727,603	0.124
Beta 2	-1,777,595	-1,777,499	0.126

Taking a closer look into the predictive statistics (RMSE), for Phase II, the UL model showed 1,221 (4%) of out-of-control observations, whereas the beta 1 model showed 15 out-of-control observations (0.0005%) and 0 (0%) out-of-control observations adopting the beta 2 model. An important effect is that the observed month in Phase II is June (transition from Autumn to Winter season), which is characterized by lower temperatures through high changes in solar radiation, wind speed, and temperature variation. Therefore, further investigations can be performed towards verification of the quality of the adjusted models considering the time frequency per minute.

In addition, Table 7 shows the computational cost required to adjust and summarize the three models, considering Phase I data. Observe that the UL model showed to be three times faster than the beta 1 model, and five times faster than the beta 2 model. Therefore, the proposed UL model-based SPC approach has an advantage over the existing beta models-based SPC approach, in terms of the computational time and space required.

**Table 7:** Computational cost (time and memory space) required for each regression model (Phase I data).

	Fit (sec)	Model Summary (sec)	RAM Memory (bytes)
UL	108	0.01	60,048
Beta 1	324	213	1,149,182,240
Beta 2	456	374.4	1,207,127,200

sec = seconds.

Furthermore, a lower Random-Access Memory (RAM) consumption is required by the flexible although simpler (i.e., with fewer parameters) UL model, allowing simple computers to process greater/large data sets. In the simulation studies, beta models took up over 20,000 times more memory space than the UL models. For the real data application, it was used a notebook with AMD Ryzen 5 3500U and 12 GB DDR4 RAM.

## 6 Conclusion

Learning structures are often used to describe association across variables. Nonetheless, inferential procedures are required to guarantee robustness on the analysis. For instance, the OLS regression, with normal assumption for the response variable (*Y*). This is not the case of proportional/rate data, which present truncated and skewed information in a bounded (0, 1) interval. Therefore, this study considered a flexible UL regression model, which can model symmetric, right- and left-asymmetric truncated unit data, despite having a single parameter.

Variables integrated in the current study were previously chosen by other authors in the study of fog/humidity distribution. [6] developed a method for calculating diurnal patterns of air temperature, wind speed, global radiation and relative humidity, and validated it with data from different countries. Some other studies, as [41], have also demonstrated the relevance of scheduling appropriately the sampling frequency of climatic variables, in order to adequately estimate land surface fluxes. A study based on solar radiation, air temperature, relative humidity and dew point [42], daily and monthly reported over a year, has revealed the minimum relative humidity coinciding with the driest month of the year.

The process of reference evapotranspiration (ET) calculation is commonly estimated through Penman-Monteith ET [43]. This equation, based on the original [44]’s equation, determines evaporation based on the combination of energy balance and aerodynamic formula; and the [45]’s modification, that includes the surface resistance denominator. Finally, the FAO adapted the formula for crops [46]. This ET estimating reference has previously used daily weather forecast [47]. The solar radiation provides energy to vaporize water and heat up the atmosphere and ground. So, a day scale monitoring for wind speed, temperature, and solar radiation was used to describe the relative humidity in the air. Accordingly, to the FAO formulation, for hourly periods, the soil heat flux (G) can be daylight periods estimated with net radiation (0.1*Rn) for night-time (0.5*Rn).

Occurrence and distribution of *Camanchaca* along northern Chile had been previously described [48–51]. It is characterized by diurnal and interannual variability with dependence on atmospheric conditions at regional and global scales [52]. It is necessary to highlight that flora and fauna distribution in the arid area is fog-dependent [53]. Therefore, this fog plays a key role in maintaining the assemblage of animal species of the ecosystem, especially during adverse climatic periods. But it also supposes an important water resource for human settlement [48].

Results from the current study agree with [54]’s study, which determined that *Camanchaca* derived from the marine inversion layer from the Atacama Desert was more persistent, though weaker, during summer months (November-March), but greater condensed and shallower in winter months, with uncharacteristically dry air and high temperatures occurring at and above 400 m above sea level. The authors explained that the stability of the temperature inversion depends on a seasonal consistent high-humidity, onshore breeze. On the other hand, diurnal variations in wind speed and direction and moisture content and temperature, show that, during summer, there is almost no offshore breeze and that the humidity of the air mass over that site is nearly constant. However, the land-sea breeze cycle is enhanced in winter, in a way that there is considerable diurnal variation in specific humidity, correlated with the night-time breeze from the inland desert. But when night falls, wind begins to blow from the east, which lowers the atmospheric humidity. At day break, winds shift to the west and humidity rises as marine air moves east. In other words, winter-fog is more intense and shallower, in comparison to summer-fog.

It is important to highlight that, as a model, it is subjected to limitations. So, further statistical models should include some interannual variations or distinguish patterns affected by the climatic conditions, such as the ENSO (El Niño-Southern Oscilation); for example, La Niña conditions promote a lower cloud amount [52]. In this manner, SPC models can assess the weather monitoring, whenever its suppositions are carefully adopted. Further studies shall explore more the data structure dependence in the statistical inference procedure, such as spatial-temporal memory.

## Supporting information

S1 Appendix Graphical visualization of simulation results. (PDF)

S2 Appendix Beta regression models’ estimation results and control charts. (PDF)
