# Supplementary material for: Unraveling water monitoring association towards weather attributes for response proportions data: A unit-Lindley learning
Source: PLoS One. 2022 Oct 14;17(10):e0275841. doi: 10.1371/journal.pone.0275841 (PMC9565758; doi:10.1371/journal.pone.0275841)
Supplement: S1 Appendix — (PDF) [file pone.0275841.s001.pdf]

S1 Appendix - Graphical visualization of simulation results

This appendix describes the data-generating process to develop further analyses towards the UL distribution robustness, for various n, $\text{ARL}_0 = \{10, 100\}$, as well adopting the β structure variation. The graphics shown in Figs 10-45 present the estimation for three different $\mu = \{0.2, 0.5, 0.8\}$, the ARL_0 and ARL_1, MRL_0 and MRL_1, SDRL_0 and SDRL_1, varying the synthetic data size $n = \{100, 200, 500, 1{,}000\}$, and considering $\alpha = \{0.1, 0.01\}$. Specifically, for $\alpha = 0.1$, we have that $\text{ARL}_0 = 10$, $\text{MRL}_0 \approx 6.579$ and $\text{SDRL}_0 \approx 9.487$; whereas for $\alpha = 0.01$, it follows that $\text{ARL}_0 = 100$, $\text{MRL}_0 \approx 68.968$ and $\text{SDRL}_0 \approx 99.499$. In all figures, these nominal (or target) values of the in-control measures (ARL_0, MRL_0 and SDRL_0) are shown exactly at the center of the graphics. The other three lower values and three upper values of μ, identified in these graphics, correspond to changes (decreases and increases, respectively) in the process mean, that is, they are related to the out-of-control measures (ARL_1, MRL_1 and SDRL_1), for which small values are desirable. Moreover, in all graphics, the continuous line with filled circle corresponds to the UL regression control chart, whereas the continuous line with triangle symbol relates to the beta regression control chart.

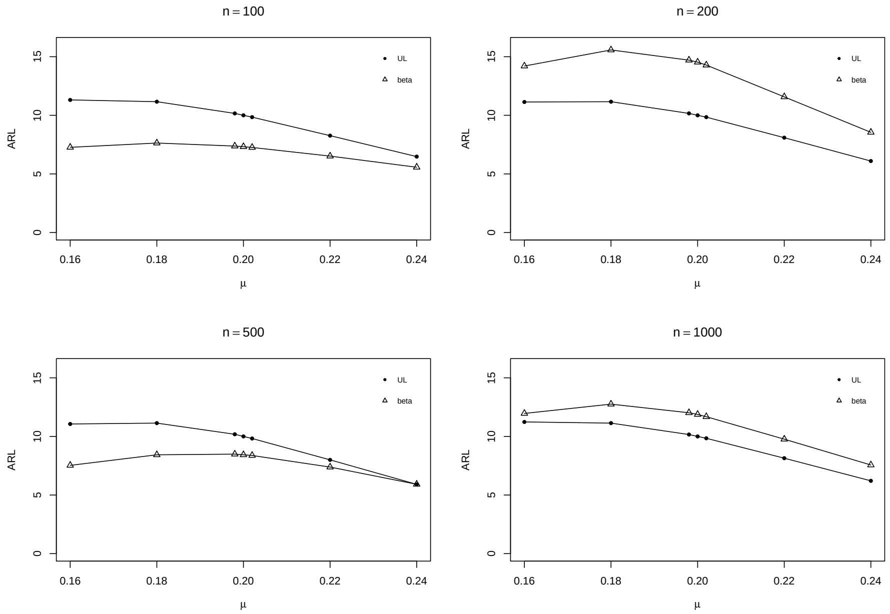

Fig 10. The ARL_0 and ARL_1 values when the true data-generating process is UL distributed, for various n ($\mu = 0.2$ and $\alpha = 0.1$).

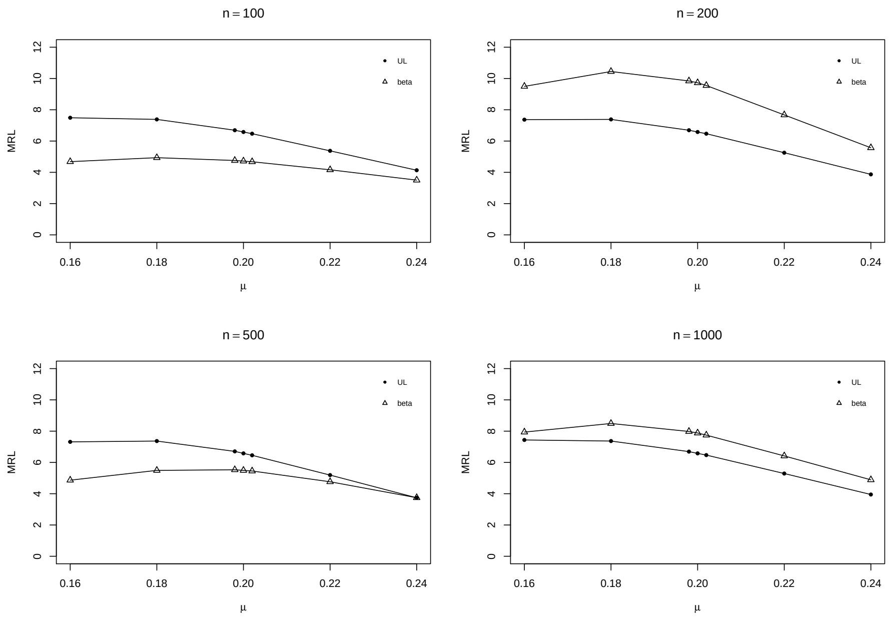

Fig 11. The MRL_0 and MRL_1 values when the true data-generating process is UL distributed, for various n ($\mu = 0.2$ and $\alpha = 0.1$).

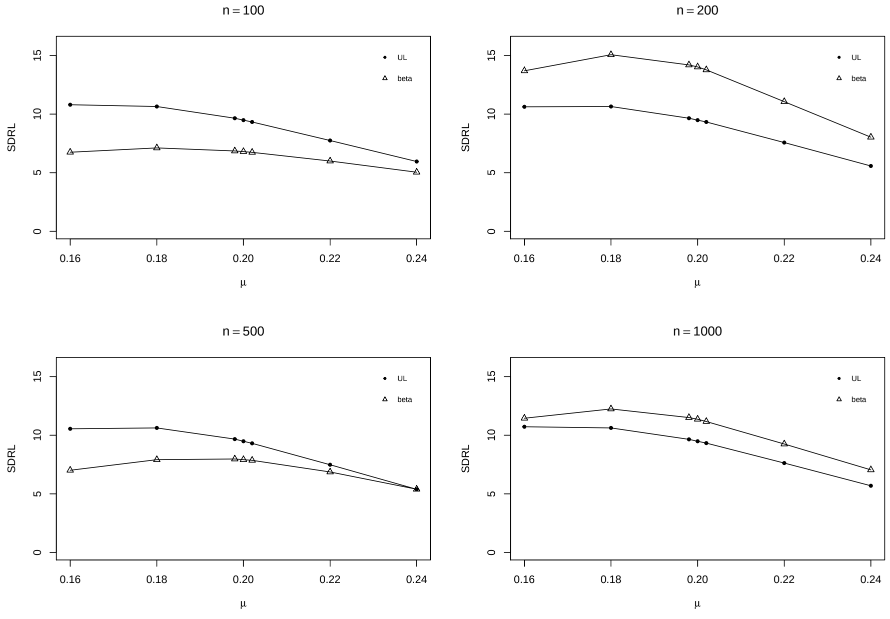

Fig 12. The SDRL_0 and SDRL_1 values when the true data-generating process is UL distributed, for various n ($\mu = 0.2$ and $\alpha = 0.1$).

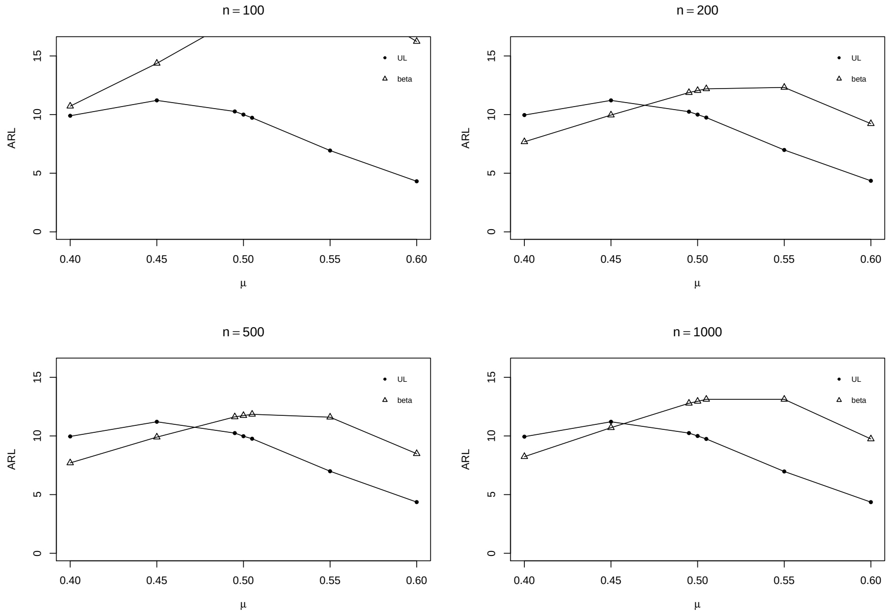

Fig 13. **The $\mathbf{ARL_0}$ and $\mathbf{ARL_1}$ values when the true data-generating process is UL distributed, for various n ($\mu = 0.5$ and $\alpha = 0.1$).**

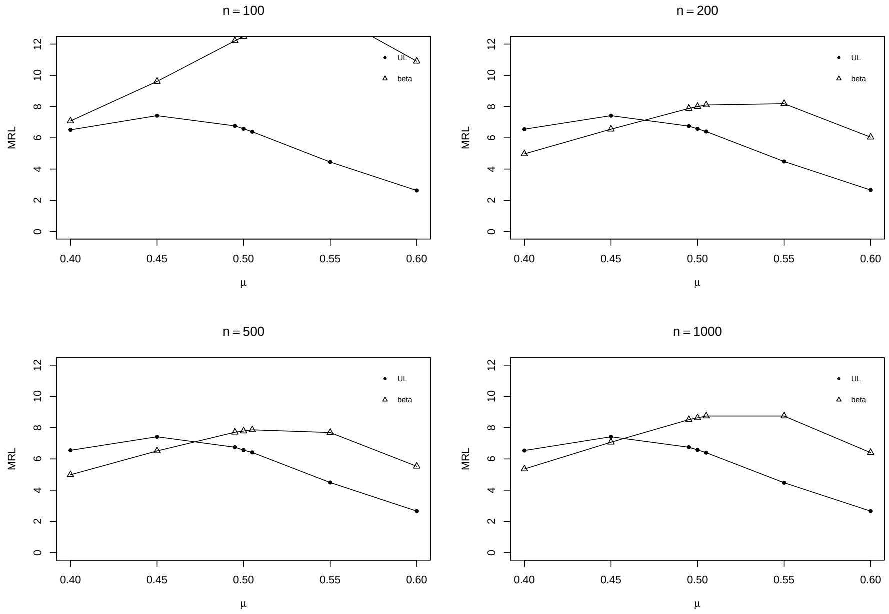

Fig 14. **The $\mathbf{MRL_0}$ and $\mathbf{MRL_1}$ values when the true data-generating process is UL distributed, for various n ($\mu = 0.5$ and $\alpha = 0.1$).**

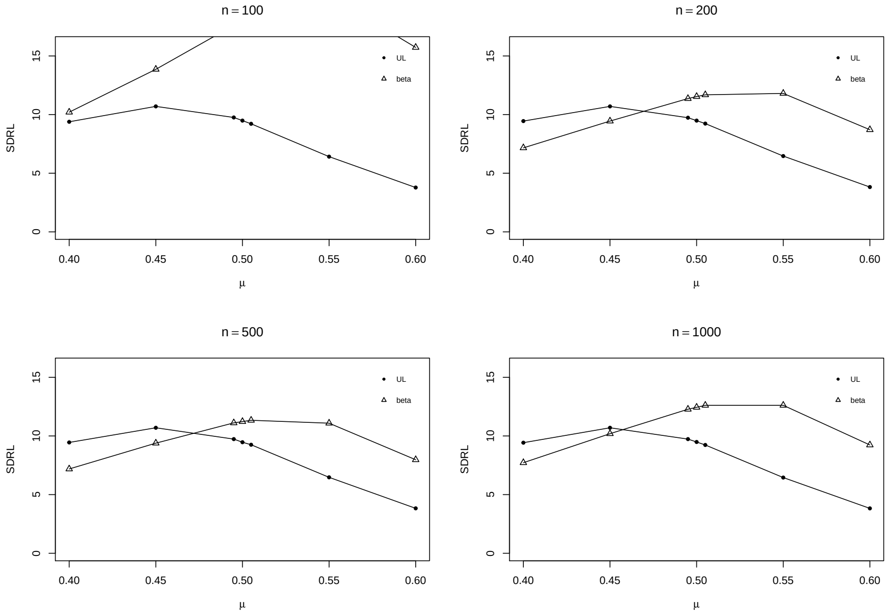

Fig 15. The $\mathbf{SDRL_0}$ and $\mathbf{SDRL_1}$ values when the true data-generating process is UL distributed, for various n ($\mu = 0.5$ and $\alpha = 0.1$).

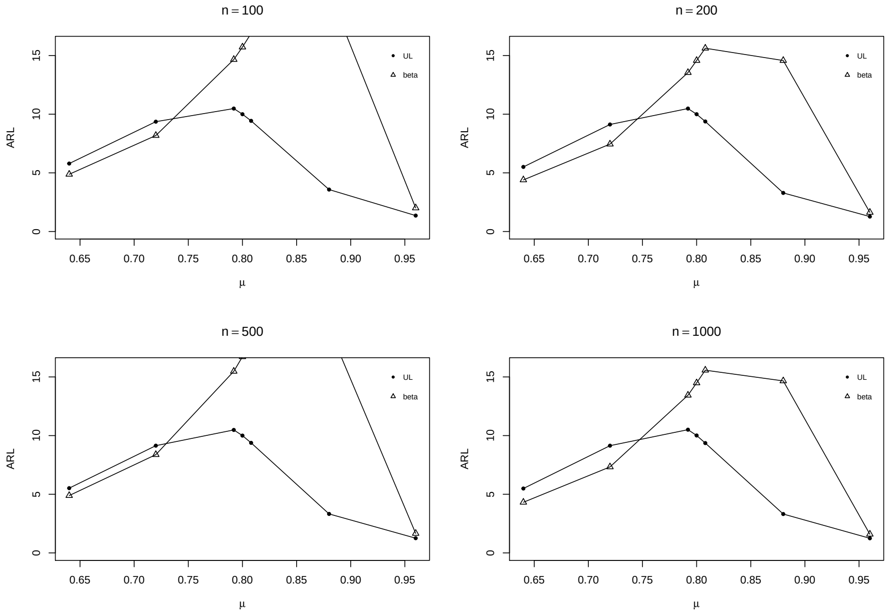

Fig 16. The $\mathbf{ARL_0}$ and $\mathbf{ARL_1}$ values when the true data-generating process is UL distributed, for various n ($\mu = 0.8$ and $\alpha = 0.1$).

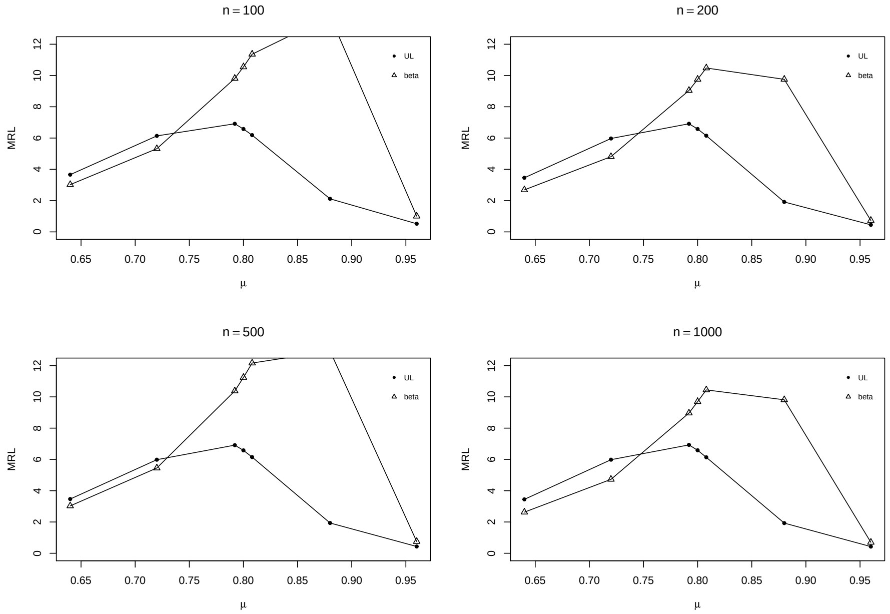

Fig 17. The MRL_0 and MRL_1 values when the true data-generating process is UL distributed, for various n ($\mu = 0.8$ and $\alpha = 0.1$).

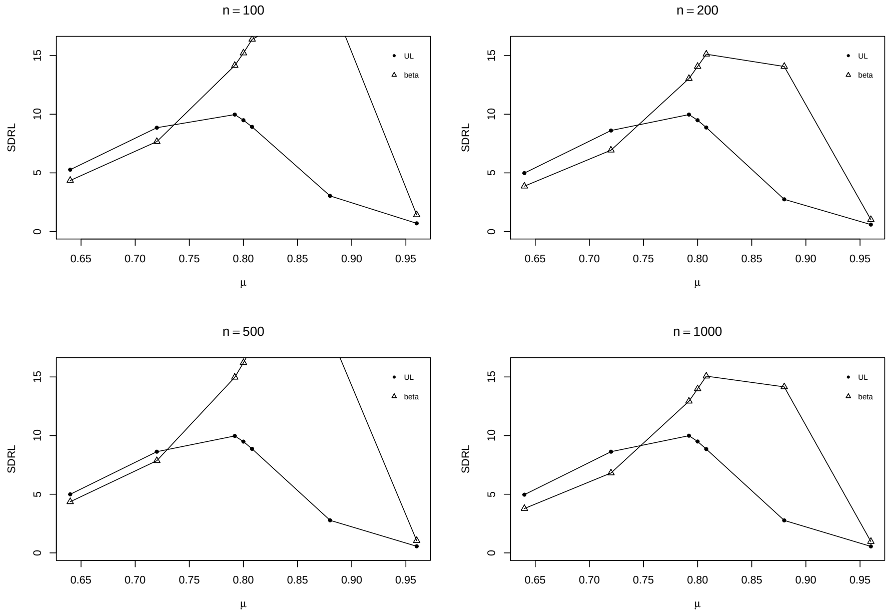

Fig 18. The $SDRL_0$ and $SDRL_1$ values when the true data-generating process is UL distributed, for various n ($\mu = 0.8$ and $\alpha = 0.1$).

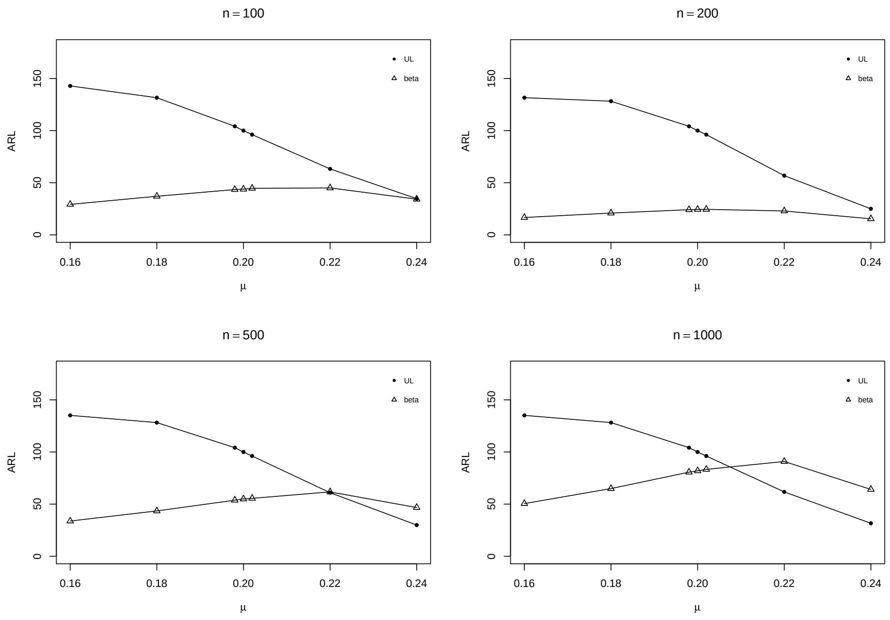

Fig 19. The $\mathbf{ARL_0}$ and $\mathbf{ARL_1}$ values when the true data-generating process is UL distributed, for various n ($\mu = 0.2$ and $\alpha = 0.01$).

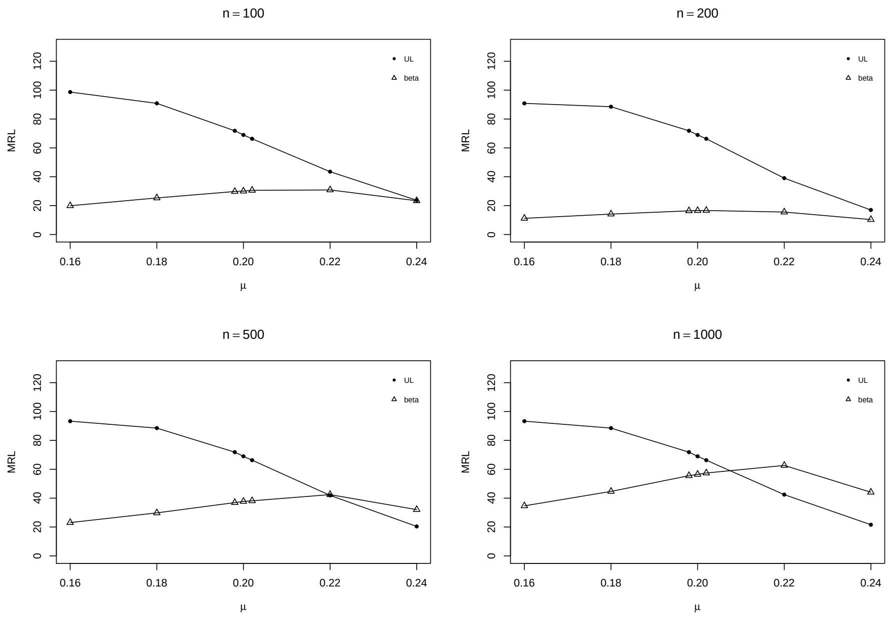

Fig 20. The $\mathbf{MRL_0}$ and $\mathbf{MRL_1}$ values when the true data-generating process is UL distributed, for various n ($\mu = 0.2$ and $\alpha = 0.01$).

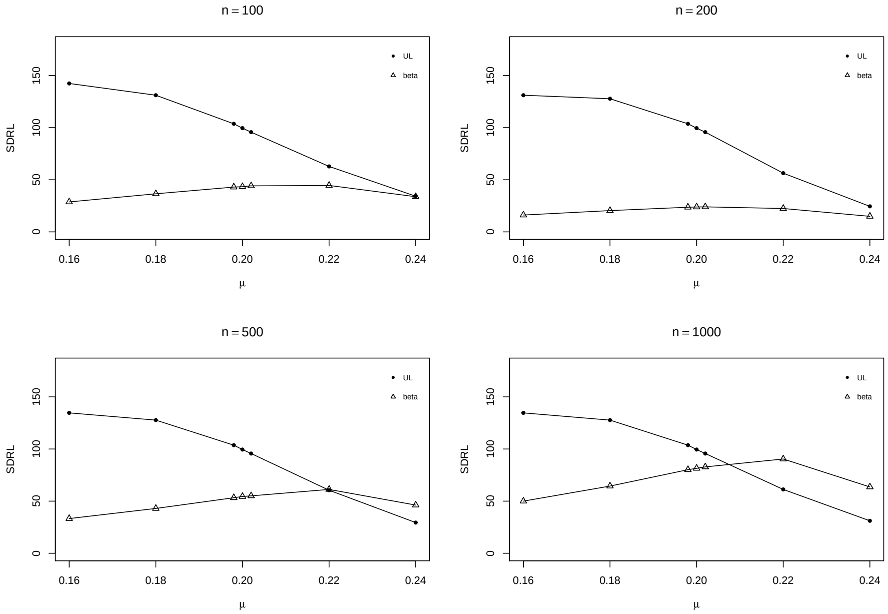

Fig 21. The SDRL_0 and SDRL_1 values when the true data-generating process is UL distributed, for various n ($\mu = 0.2$ and $\alpha = 0.01$).

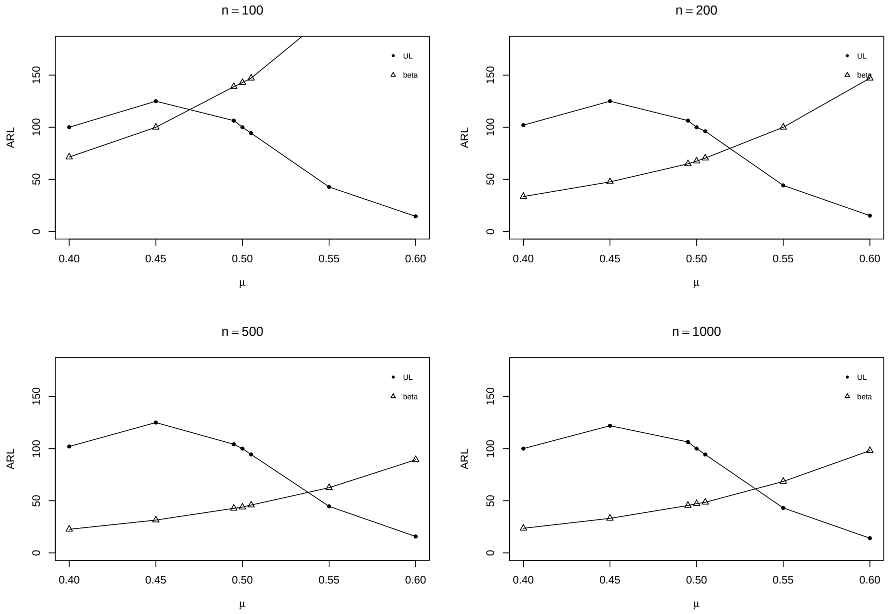

Fig 22. The ARL_0 and ARL_1 values when the true data-generating process is UL distributed, for various n ($\mu = 0.5$ and $\alpha = 0.01$).

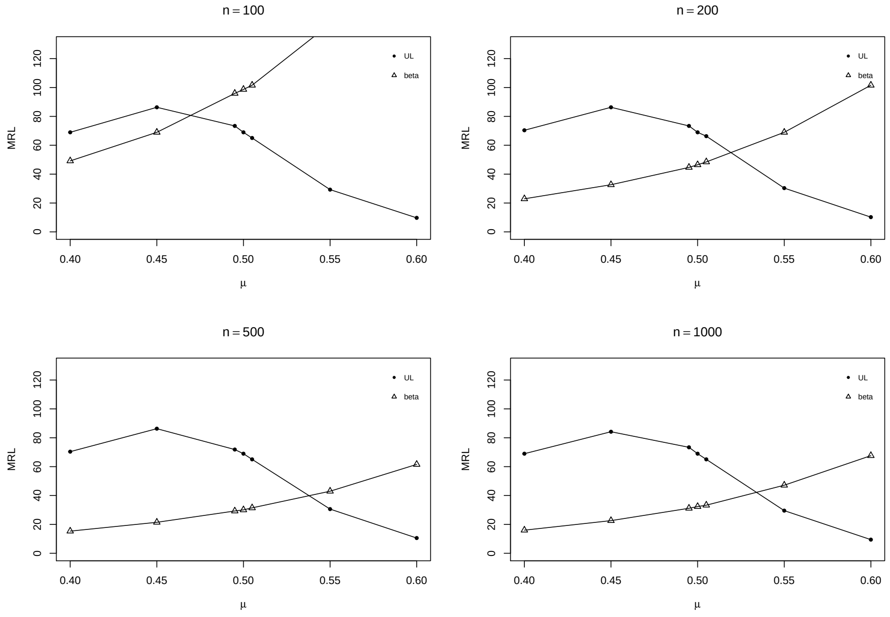

Fig 23. The MRL_0 and MRL_1 values when the true data-generating process is UL distributed, for various n ($\mu = 0.5$ and $\alpha = 0.01$).

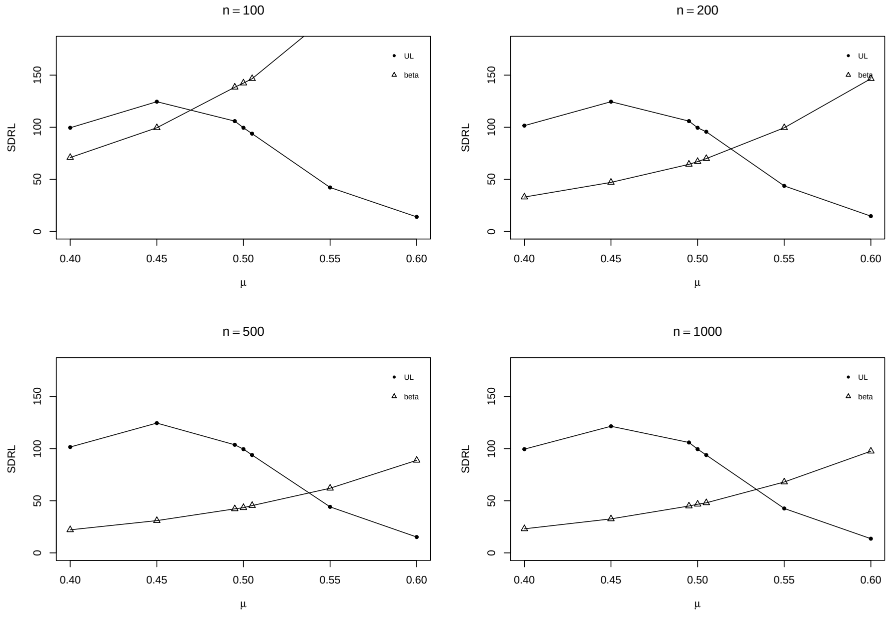

Fig 24. The $SDRL_0$ and $SDRL_1$ values when the true data-generating process is UL distributed, for various n ($\mu = 0.5$ and $\alpha = 0.01$).

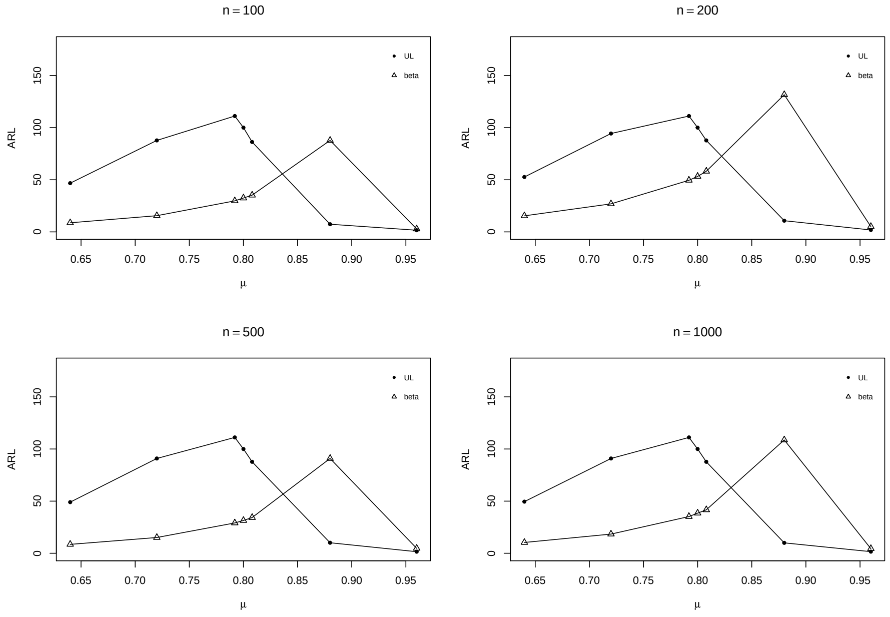

Fig 25. The ARL_0 and ARL_1 values when the true data-generating process is UL distributed, for various n ($\mu = 0.8$ and $\alpha = 0.01$).

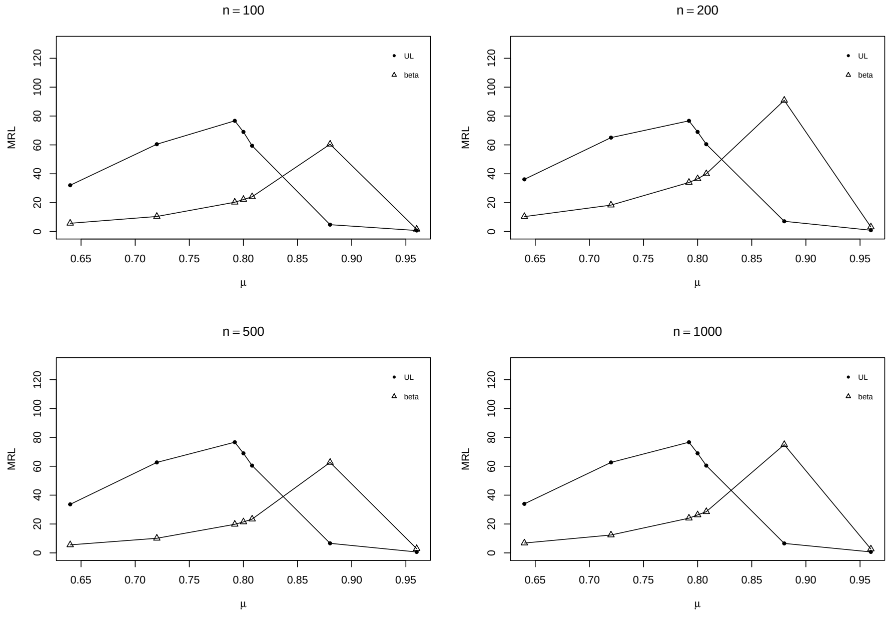

Fig 26. The MRL_0 and MRL_1 values when the true data-generating process is UL distributed, for various n ($\mu = 0.8$ and $\alpha = 0.01$).

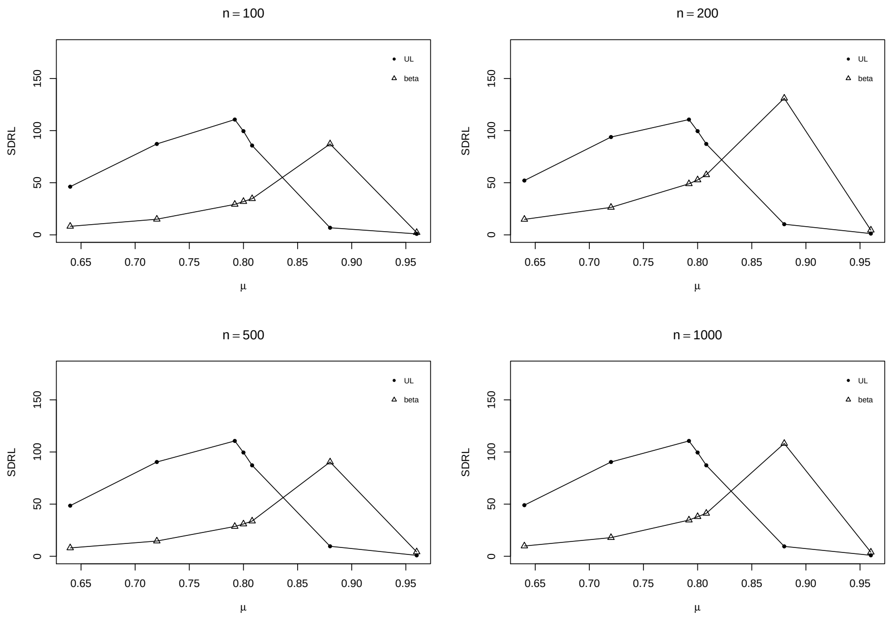

Fig 27. The $\mathbf{SDRL_0}$ and $\mathbf{SDRL_1}$ values when the true data-generating process is UL distributed, for various n ($\mu = 0.8$ and $\alpha = 0.01$).

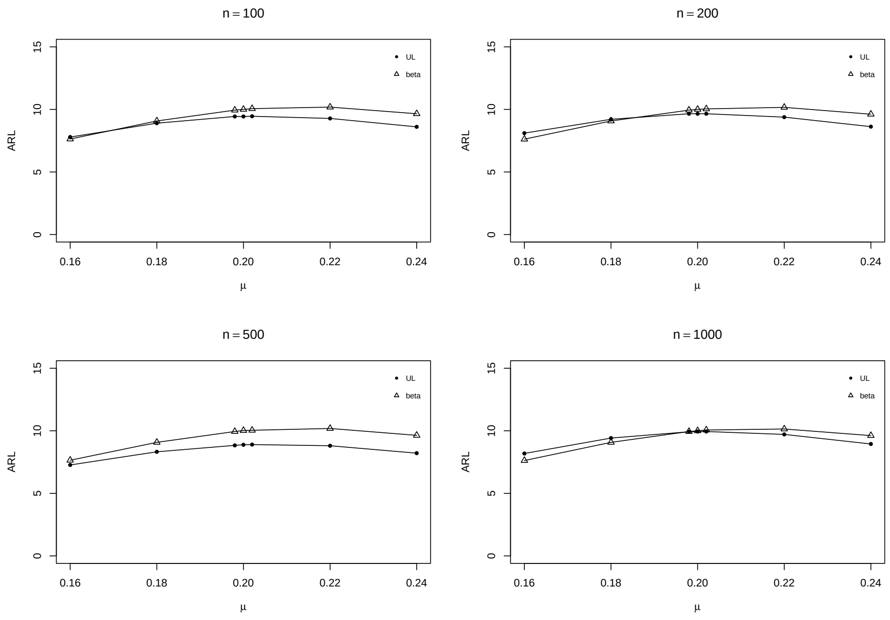

Fig 28. The $\mathbf{ARL_0}$ and $\mathbf{ARL_1}$ values when the true data-generating process is beta distributed, for various n ($\mu = 0.2$ and $\alpha = 0.1$).

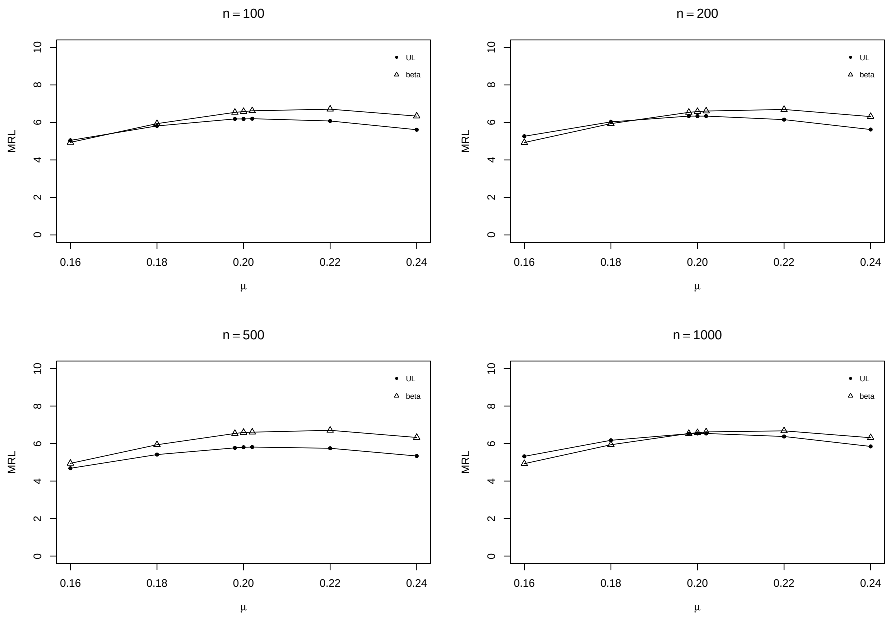

Fig 29. The MRL_0 and MRL_1 values when the true data-generating process is beta distributed, for various n ($\mu = 0.2$ and $\alpha = 0.1$).

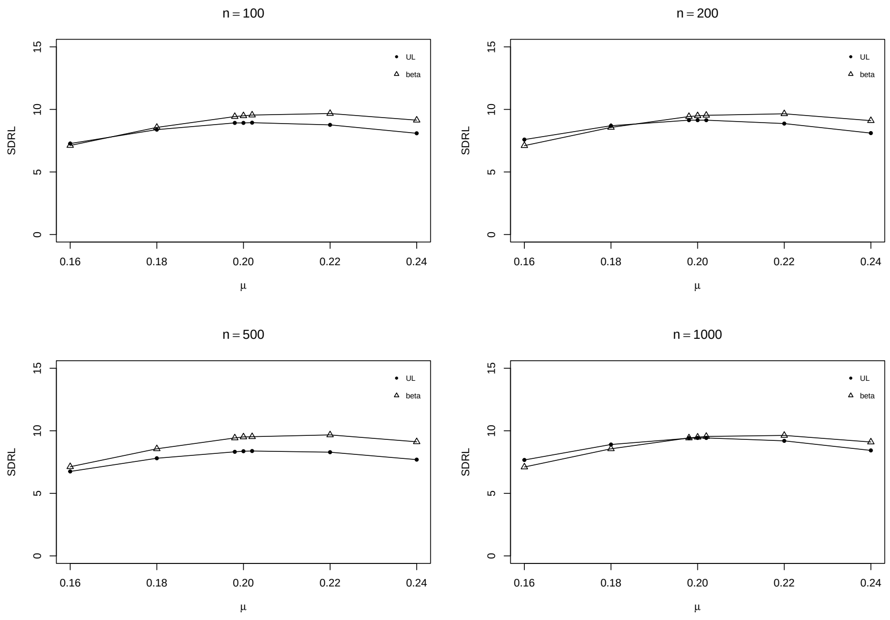

Fig 30. The $SDRL_0$ and $SDRL_1$ values when the true data-generating process is beta distributed, for various n ($\mu = 0.2$ and $\alpha = 0.1$).

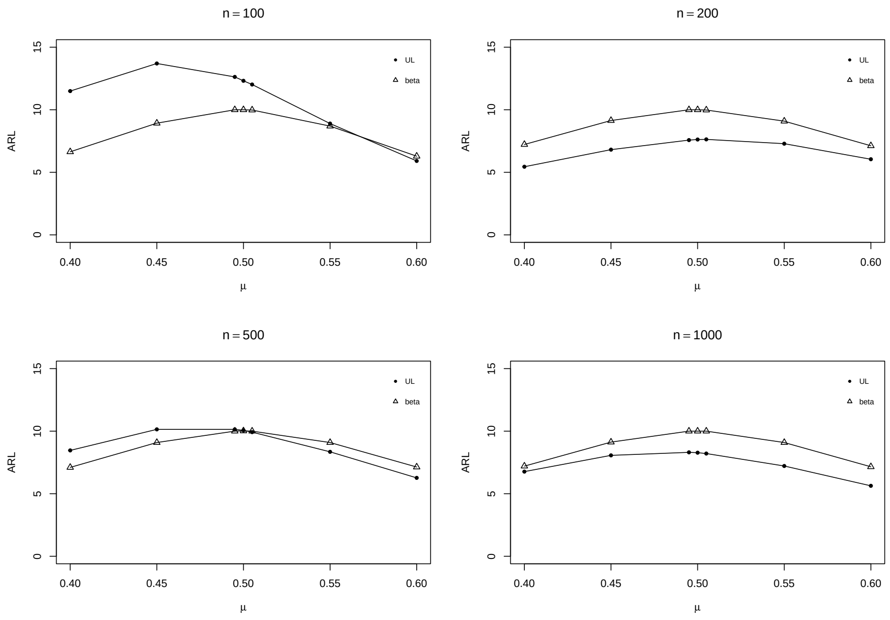

Fig 31. The ARL_0 and ARL_1 values when the true data-generating process is beta distributed, for various n ($\mu = 0.5$ and $\alpha = 0.1$).

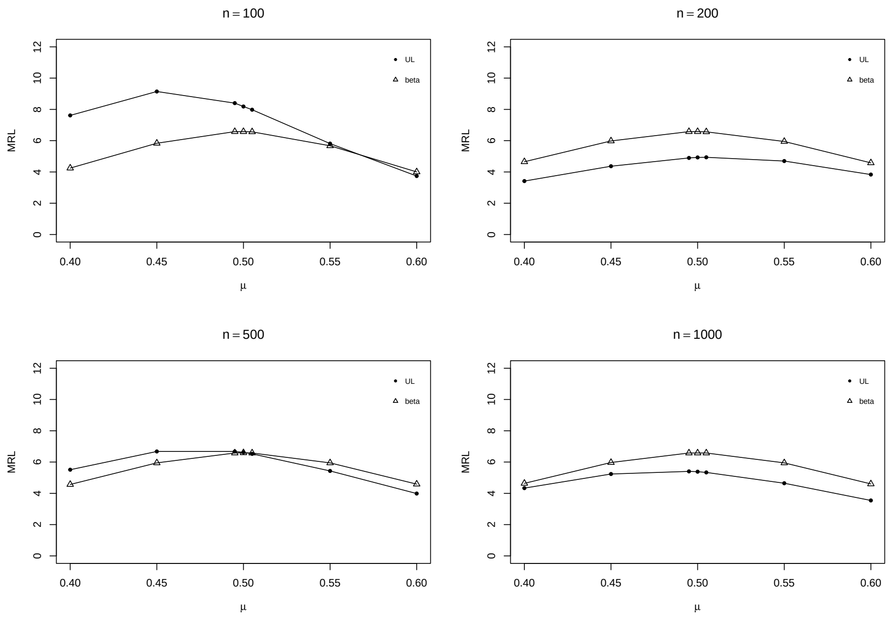

Fig 32. The MRL_0 and MRL_1 values when the true data-generating process is beta distributed, for various n ($\mu = 0.5$ and $\alpha = 0.1$).

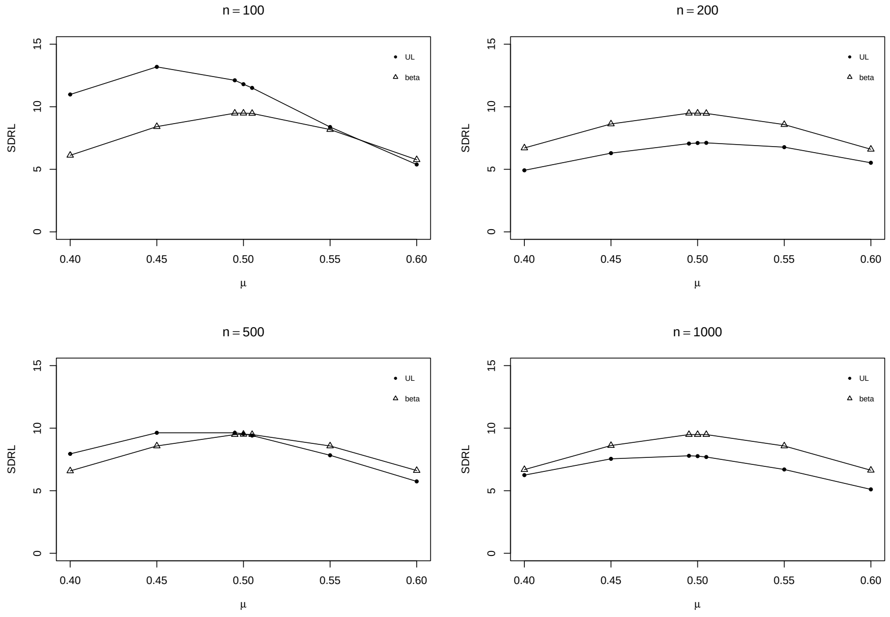

Fig 33. The $SDRL_0$ and $SDRL_1$ values when the true data-generating process is beta distributed, for various n ($\mu = 0.5$ and $\alpha = 0.1$).

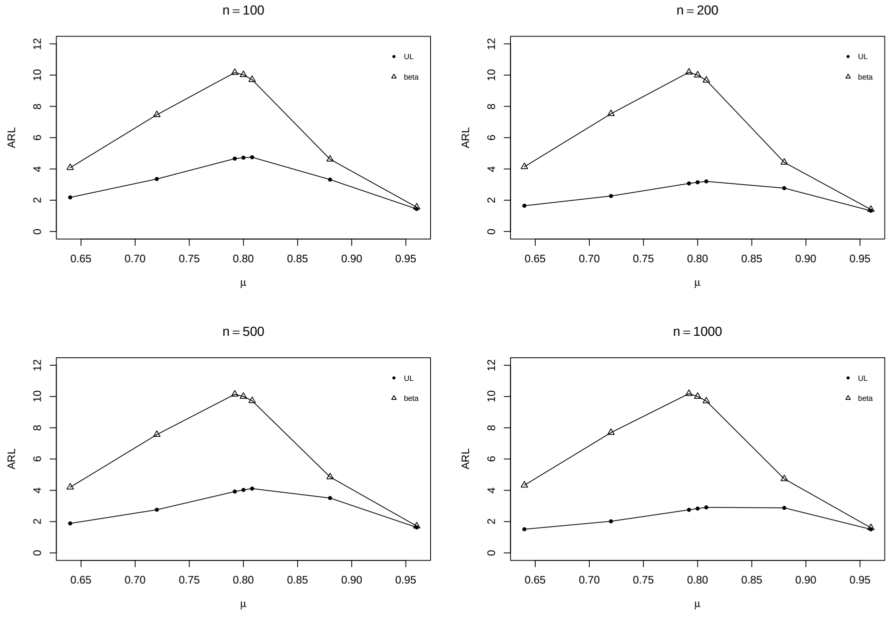

Fig 34. The ARL_0 and ARL_1 values when the true data-generating process is beta distributed, for various n ($\mu = 0.8$ and $\alpha = 0.1$).

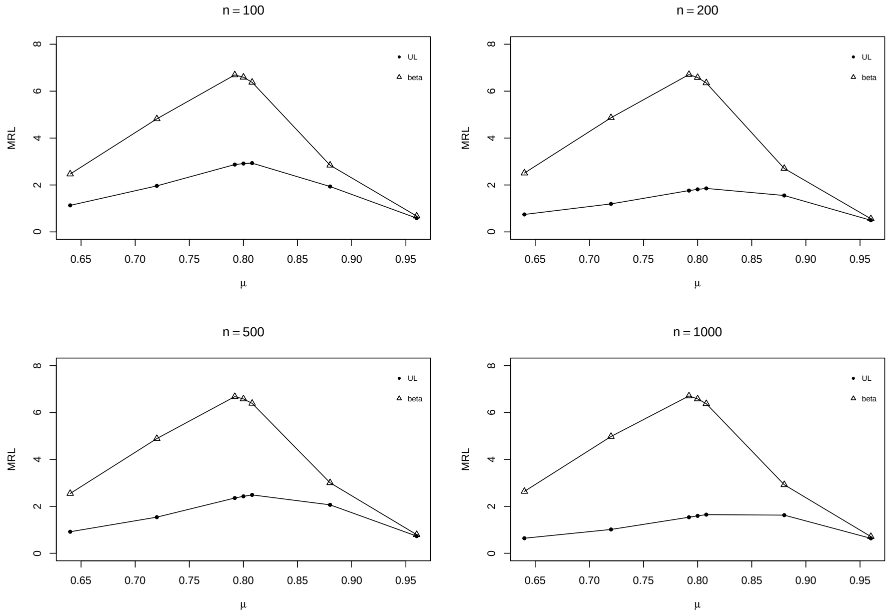

Fig 35. The MRL_0 and MRL_1 values when the true data-generating process is beta distributed, for various n ($\mu = 0.8$ and $\alpha = 0.1$).

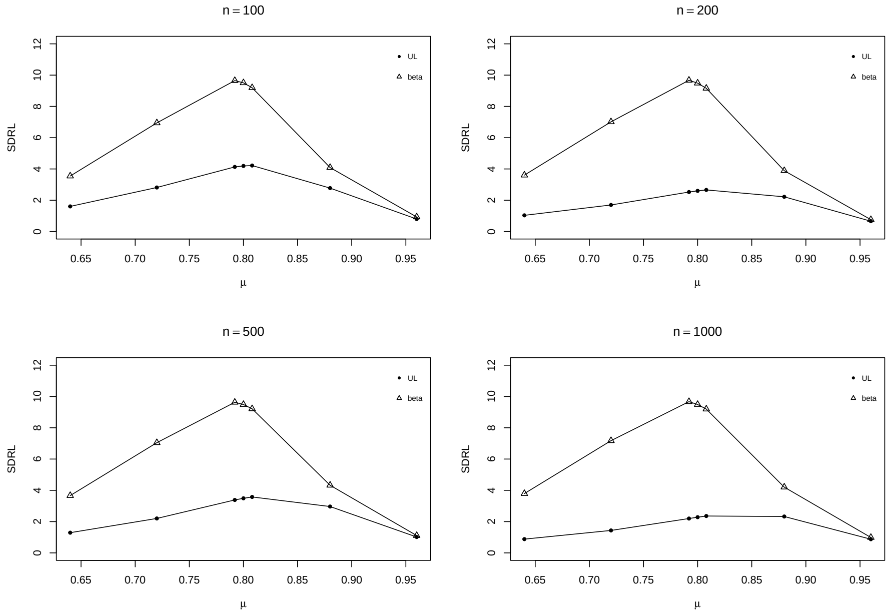

Fig 36. The SDRL_0 and SDRL_1 values when the true data-generating process is beta distributed, for various n ($\mu = 0.8$ and $\alpha = 0.1$).

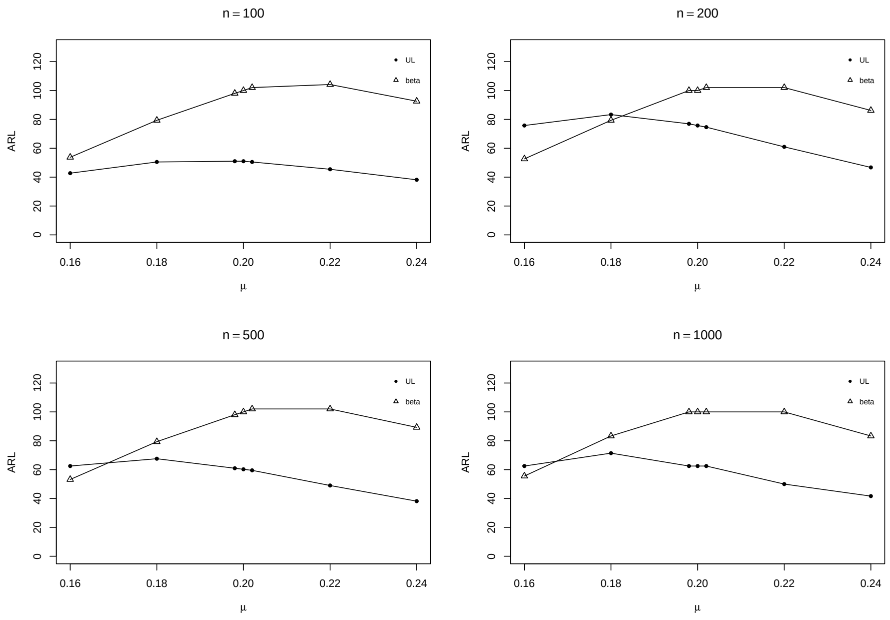

Fig 37. The ARL_0 and ARL_1 values when the true data-generating process is beta distributed, for various n ($\mu = 0.2$ and $\alpha = 0.01$).

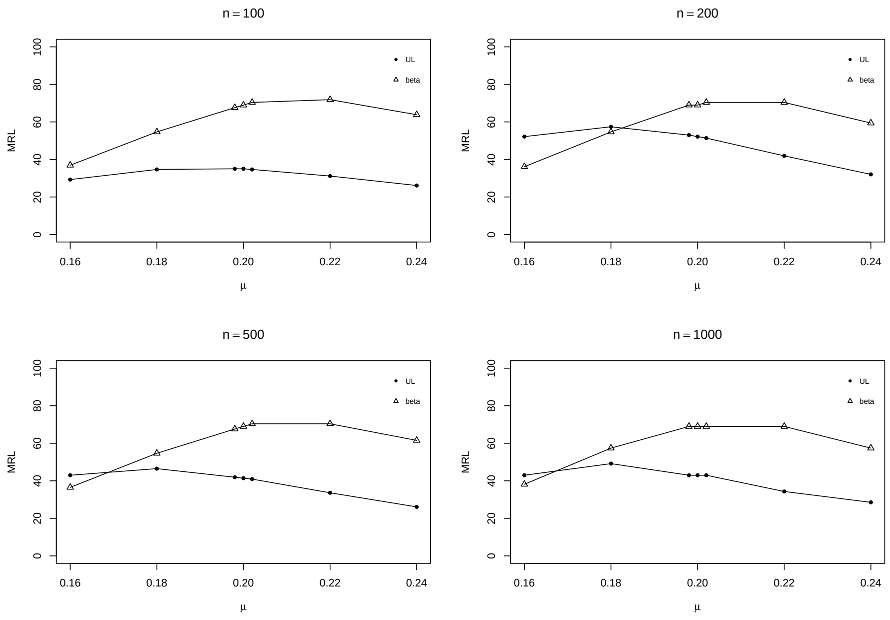

Fig 38. The MRL_0 and MRL_1 values when the true data-generating process is beta distributed, for various n ($\mu = 0.2$ and $\alpha = 0.01$).

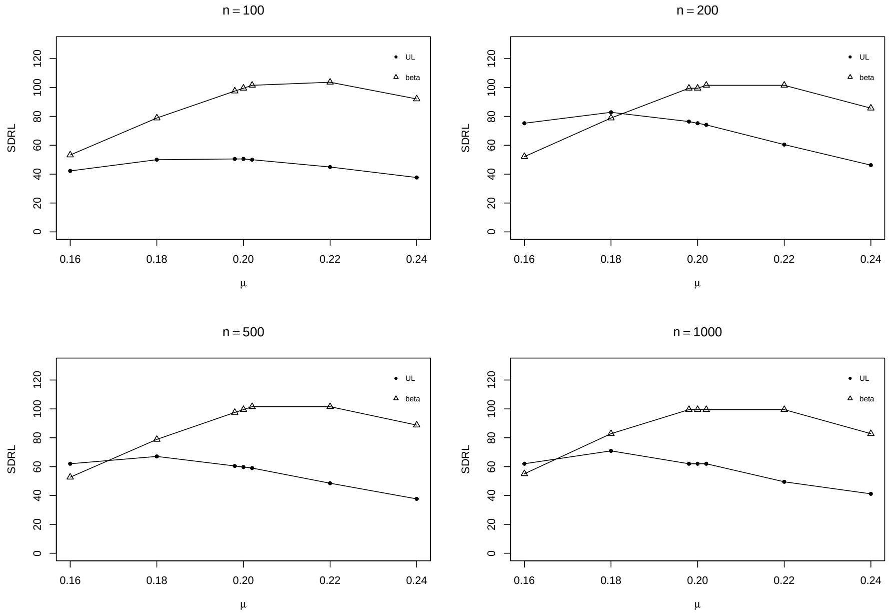

Fig 39. The SDRL_0 and SDRL_1 values when the true data-generating process is beta distributed, for various n ($\mu = 0.2$ and $\alpha = 0.01$).

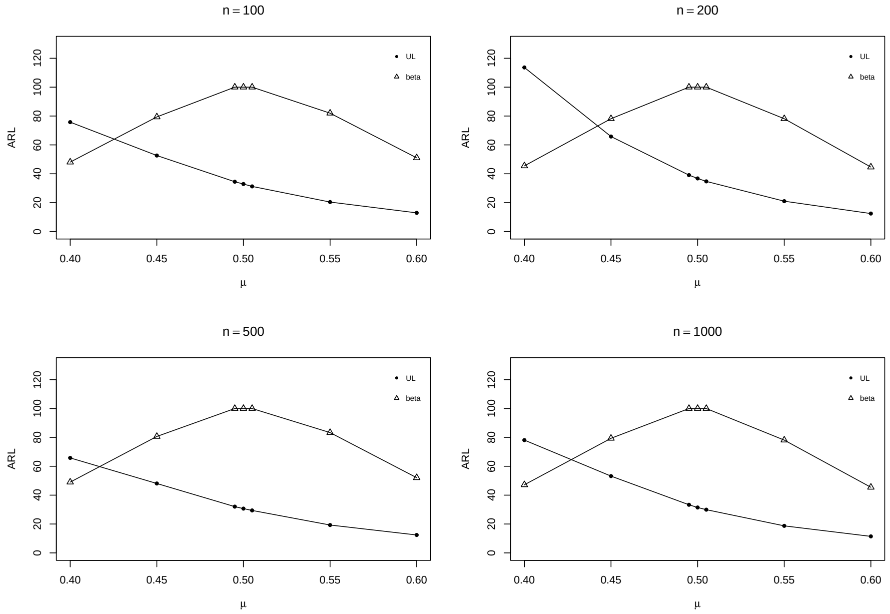

Fig 40. The ARL_0 and ARL_1 values when the true data-generating process is beta distributed, for various n ($\mu = 0.5$ and $\alpha = 0.01$).

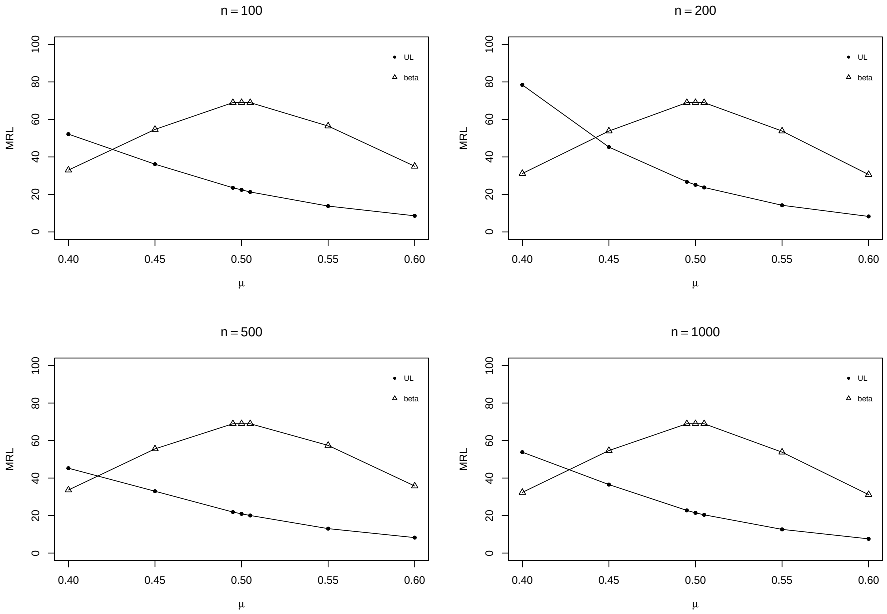

Fig 41. The MRL_0 and MRL_1 values when the true data-generating process is beta distributed, for various n ($\mu = 0.5$ and $\alpha = 0.01$).

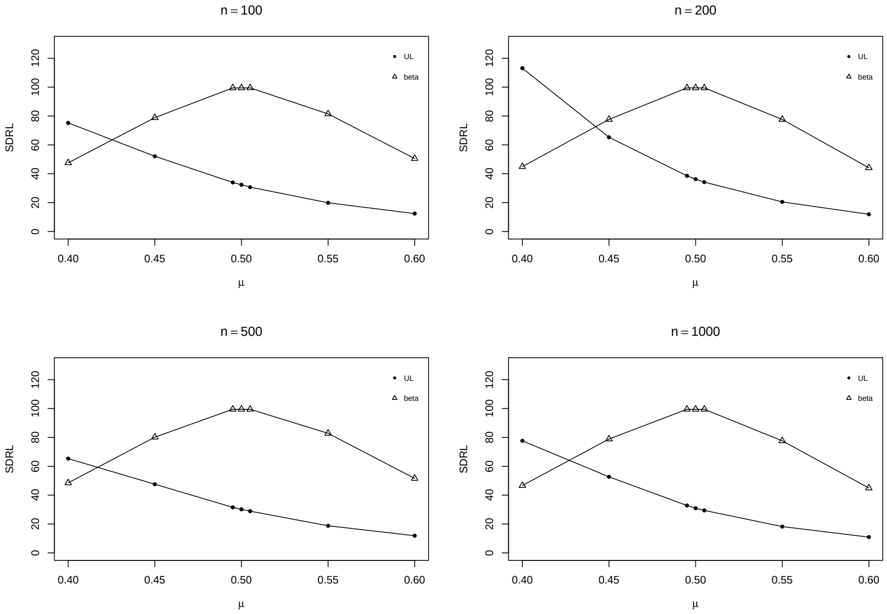

Fig 42. The SDRL_0 and SDRL_1 values when the true data-generating process is beta distributed, for various n ($\mu = 0.5$ and $\alpha = 0.01$).

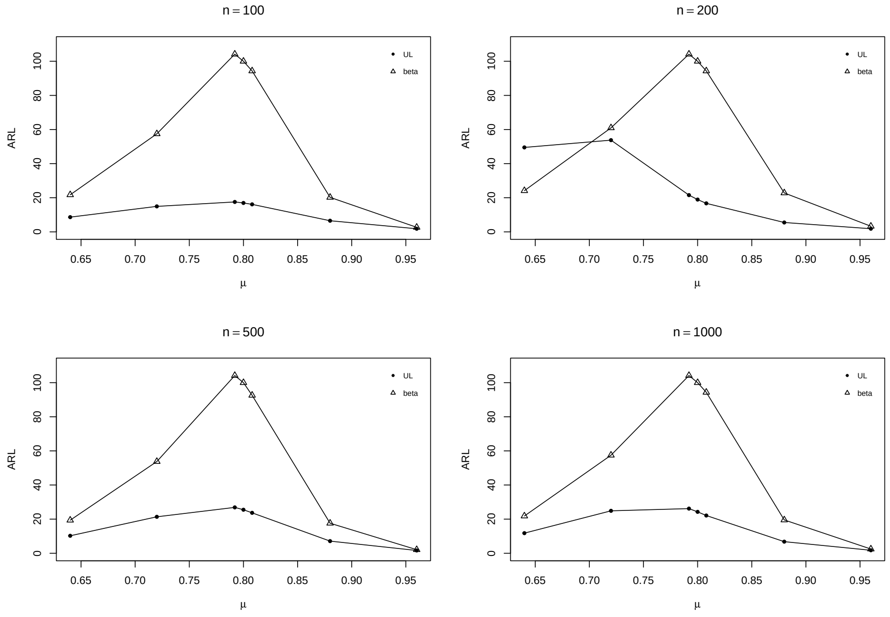

Fig 43. The ARL_0 and ARL_1 values when the true data-generating process is beta distributed, for various n ($\mu = 0.8$ and $\alpha = 0.01$).

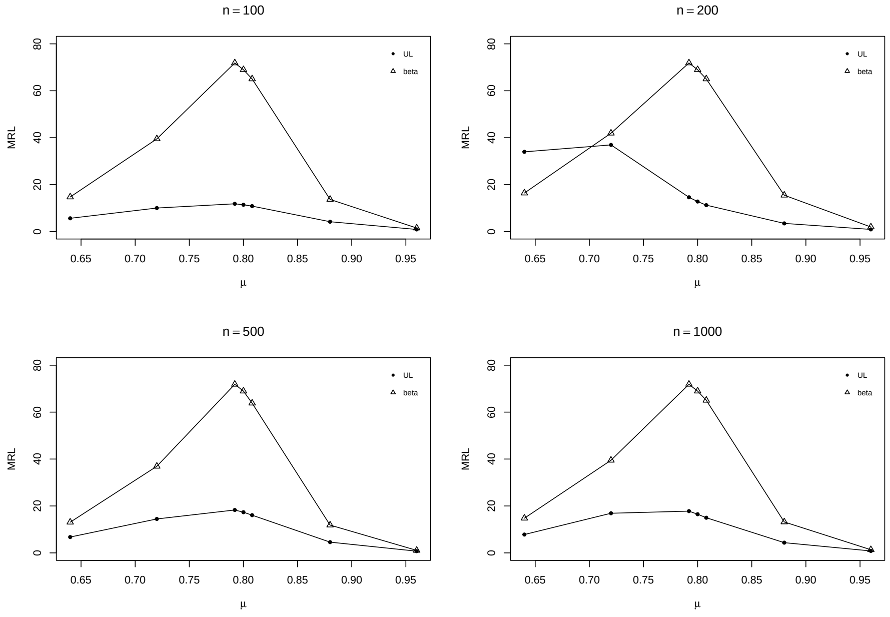

Fig 44. The MRL_0 and MRL_1 values when the true data-generating process is beta distributed, for various n ($\mu = 0.8$ and $\alpha = 0.01$).

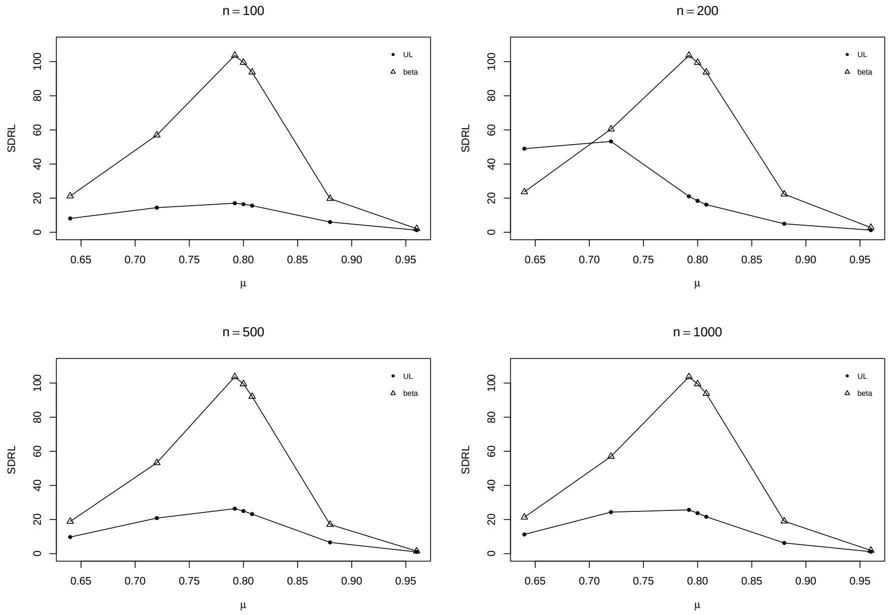

Fig 45. The SDRL_0 and SDRL_1 values when the true data-generating process is beta distributed, for various n ($\mu = 0.8$ and $\alpha = 0.01$).
