# Supplementary material for: Unraveling water monitoring association towards weather attributes for response proportions data: A unit-Lindley learning
Source: PLoS One. 2022 Oct 14;17(10):e0275841. doi: 10.1371/journal.pone.0275841 (PMC9565758; doi:10.1371/journal.pone.0275841)
Supplement: S2 Appendix — (PDF) [file pone.0275841.s002.pdf]

S2 Appendix - Beta regression models' estimation results and control charts

This appendix provides the ML estimation results for the beta regression model with constant and varying dispersion (beta 1 and beta 2 models, respectively) applied to the relative humidity data, as well as the obtained control charts (beta 1 and beta 2 control charts).

Table 8. Beta 1 model adjusted for relative humidity data.

	Estimate	Std. error	t stat	p-value
	**Mean submodel**			
Intercept	0.6393	0.0016	407.7	< 0.0001
Wind Speed	-0.0795	0.0002	-373.4	< 0.0001
ΔTemperature	0.0838	0.0001	624.7	< 0.0001
Solar Radiation	-0.0017	< 0.0001	-679.9	< 0.0001
	**Dispersion submodel**			
Intercept	-0.9672	0.0008	-1,206.0	< 0.0001

Table 9. Beta 2 model adjusted for relative humidity data.

	Estimate	Std. error	t stat	p-value
	**Mean submodel**			
Intercept	0.8034	0.0021	383.3	< 0.0001
Wind Speed	-0.0775	0.0002	-355.5	< 0.0001
ΔTemperature	0.0653	0.0002	351.8	< 0.0001
Solar Radiation	-0.0011	< 0.0001	-704.3	< 0.0001
	**Dispersion submodel**			
Intercept	-0.6562	0.0027	-238.4	< 0.0001
Wind Speed	-0.0695	0.0303	100.8	< 0.0001
ΔTemperature	-0.0456	0.0002	-179.7	< 0.0001
Solar Radiation	-0.0002	< 0.0001	-165.6	< 0.0001

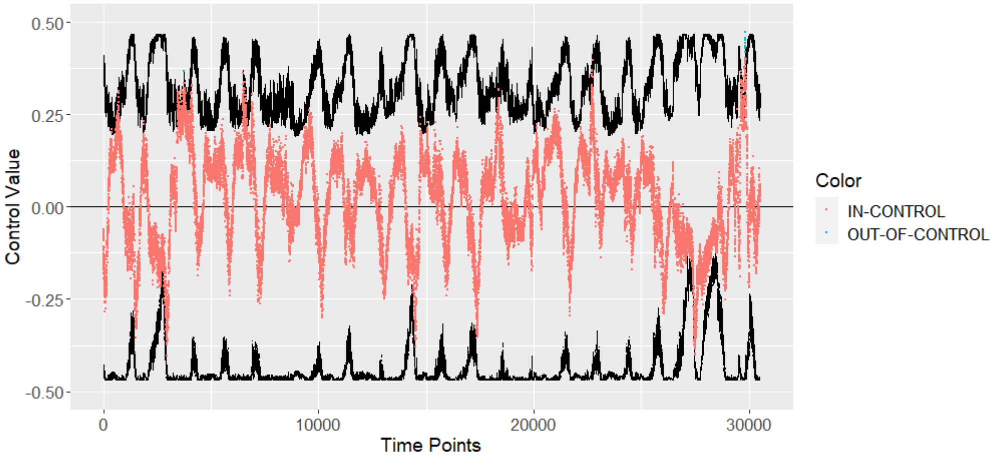

Fig 46. Beta 1 control chart (Phase II) adopting the residual representation.

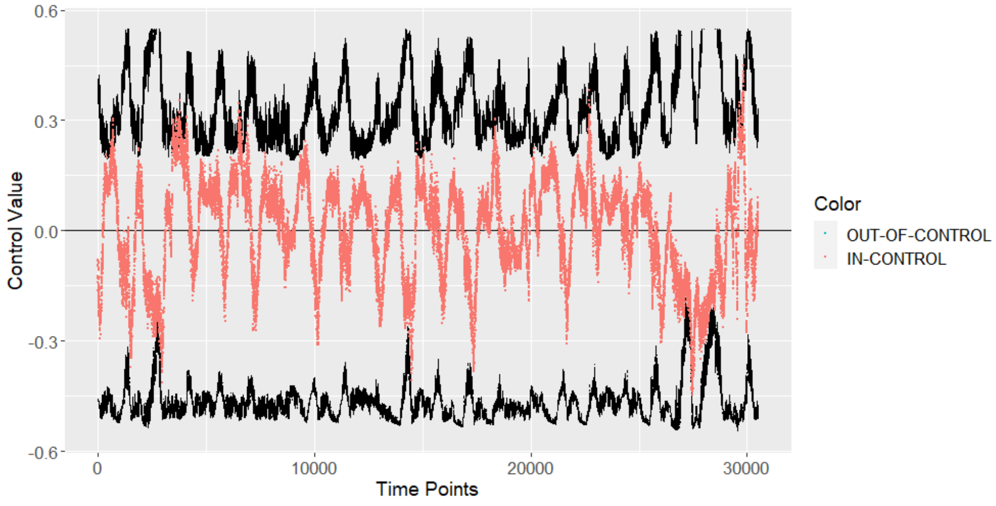

Fig 47. Beta 2 control chart (Phase II) adopting the residual representation.
